# Bacterial Communities of Diverse *Drosophila* Species: Ecological Context of a Host–Microbe Model System

**DOI:** 10.1371/journal.pgen.1002272

**Published:** 2011-09-22

**Authors:** James Angus Chandler, Jenna Morgan Lang, Srijak Bhatnagar, Jonathan A. Eisen, Artyom Kopp

**Affiliations:** 1 Center for Population Biology, Department of Evolution and Ecology, University of California Davis, Davis, California, United States of America; 2 UC Davis Genome Center, University of California Davis, Davis, California, United States of America; 3 Department of Medical Microbiology and Immunology, School of Medicine, University of California Davis, Davis, California, United Stated of America; 4 United States Department of Energy Joint Genome Institute, Walnut Creek, California, United States of America; Fred Hutchinson Cancer Research Center, United States of America

## Abstract

*Drosophila melanogaster* is emerging as an important model of non-pathogenic host–microbe interactions. The genetic and experimental tractability of *Drosophila* has led to significant gains in our understanding of animal–microbial symbiosis. However, the full implications of these results cannot be appreciated without the knowledge of the microbial communities associated with natural *Drosophila* populations. In particular, it is not clear whether laboratory cultures can serve as an accurate model of host–microbe interactions that occur in the wild, or those that have occurred over evolutionary time. To fill this gap, we characterized natural bacterial communities associated with 14 species of *Drosophila* and related genera collected from distant geographic locations. To represent the ecological diversity of Drosophilids, examined species included fruit-, flower-, mushroom-, and cactus-feeders. In parallel, wild host populations were compared to laboratory strains, and controlled experiments were performed to assess the importance of host species and diet in shaping bacterial microbiome composition. We find that Drosophilid flies have taxonomically restricted bacterial communities, with 85% of the natural bacterial microbiome composed of only four bacterial families. The dominant bacterial taxa are widespread and found in many different host species despite the taxonomic, ecological, and geographic diversity of their hosts. Both natural surveys and laboratory experiments indicate that host diet plays a major role in shaping the *Drosophila* bacterial microbiome. Despite this, the internal bacterial microbiome represents only a highly reduced subset of the external bacterial communities, suggesting that the host exercises some level of control over the bacteria that inhabit its digestive tract. Finally, we show that laboratory strains provide only a limited model of natural host–microbe interactions. Bacterial taxa used in experimental studies are rare or absent in wild *Drosophila* populations, while the most abundant associates of natural *Drosophila* populations are rare in the lab.

## Author Summary

All animals are associated with large consortia of non-pathogenic microbes. Most of these “microbiomes” are not well characterized despite their importance for many aspects of host biology including human and animal health and the agricultural impact of pest species. The fruit fly *Drosophila melanogaster* provides a powerful experimental model for investigating the dynamics and consequences of animal–microbial interactions. However, it is not clear whether the model bacteria studied in the lab are representative of natural microbial consortia. To establish an ecological and comparative background for experimental studies, we have conducted a global survey of bacterial communities associated with natural populations of 14 species of *Drosophila* and related genera. Despite the taxonomic and ecological diversity of these species, we find that they are associated with the same dominant bacterial groups. Based on our results, we propose a model of microbiome assembly where its composition is circumscribed by host diet and physiology but, within those limits, is highly dependent on chance environmental encounters. Consistent with this model, the microbiomes of wild flies differ significantly from those of laboratory strains, suggesting that experimental studies should be extended to include the bacteria that are most prevalent in natural communities.

## Introduction

The genetic and experimental tractability of *Drosophila melanogaster* often overshadows the phenotypic, evolutionary and ecological diversity of its relatives. Over 3000 species of *Drosophila* and related genera inhabit all continents except Antarctica, occur in practically every type of habitat, and show a great variety of morphological, behavioral, and life-history traits [1]. In particular, the feeding and breeding substrates vary tremendously within the Drosophilids. While the well-known cosmopolitan species are considered generalists, as decaying fruit of many different plants makes for an acceptable substrate, dietary specialization has evolved many times within *Drosophila*. A well-known example is *D. sechellia*, which specializes on the *Morinda* fruit, a resource that is toxic to most other animals [2]. Other *Drosophila* species use flowers, mushrooms, sap fluxes, cambium, decaying vegetation, and cacti as feeding and breeding sites [3], [4]. Importantly, dietary shifts have occurred numerous times within the genus, and closely related species are known to utilize different types of food sources [5], [6], [7]. At the same time, it is common to find phylogenetically distant species using the same food source. In almost all of these cases, the biotic environment that *Drosophila* are interacting with, especially the microbial communities associated with these flies, is unknown.

The importance and ubiquity of microbial associates of animals is only beginning to be appreciated. Although most attention has been devoted to pathogenic bacteria, pathogens are a small minority of animal symbionts. Bacteria can play beneficial, and often essential, roles in the lives of their hosts. In animals that carry vertically transmitted, intracellular bacteria, the host and its symbiont community form an inseparable holobiont with shared metabolism and evolutionary fate [8], [9]. However, symbionts need not be intracellular or completely dependent on the host to shape host physiology and evolution. Most animal-microbial interactions are flexible and facultative, where the symbionts can exist without the host and the host can carry different symbionts at different times. It is likely that every animal is associated with a complex and ever-changing microbial community that consists predominantly of non-pathogenic, free-living bacteria [10]. Nowhere is this more evident than in intestinal microbiology. In humans, bacterial gut fauna is composed of more than a thousand taxa and certain aspects of human health, such as obesity, are associated with an altered intestinal community [11]. Bacterial gut symbionts are equally prevalent in other mammals [12] and in insects [13], [14]. In many insects, gut symbionts are essential for survival and form the core of host physiology and ecological adaptation [15], [16], [17]. Even when not strictly essential for survival, experimental evidence suggests that insect gut fauna affects many aspects of host phenotype [18] and can mediate interactions between the host and potential pathogens [19].

The composition of bacterial symbiont communities is shaped both by host genotype and its diet. In mice and fruit flies, mutations in a single host gene can be sufficient to alter microbiome composition [20], [21]. Reciprocal transplants of intestinal microbiomes between zebrafish and mice reveal that the gut habitat of these hosts selects for different communities [22]. These differences are smaller at shorter evolutionary time scales, as species that are more closely related often share more similar bacterial communities. This trend has been observed in stinkbugs [23], termites [24], and mammals [12]. Diet also plays an important role in shaping the intestinal bacterial microbiome in many systems. When humans are shifted onto a low carbohydrate, low fat diet, their intestinal communities shift towards a higher percentage of the phylum Bacteroidetes [11]. The gut communities of European and African human populations are shaped, at least in part, by their different diets [25].

*D. melanogaster* is naturally emerging as a model of host-microbe interactions. Genetic experiments have identified some of the genes contributing to intestinal community homeostasis. The gene *PIMS* actively suppresses immune response when flies are exposed to commensal, non-pathogenic intestinal communities [26]. Similarly, downregulation of *caudal* significantly alters this bacterial community, allowing normally rare bacteria to increase in abundance [21]. However, little is known about the effects of gut bacteria on *Drosophila* physiology. Axenic strains of *D. melanogaster* are viable, at least on rich media. Although some studies suggested that gut symbionts increase life span in *D. melanogaster* [27], other studies failed to replicate this effect [21], [28]. Commensal bacteria can even affect mate choice in *D. melanogaster* in the lab [29], although the evolutionary significance of this effect in the wild is not clear.

In contrast to our increasing understanding of *Drosophila*-microbe interactions in the lab, little is known about the microbial communities associated with natural *Drosophila* populations. In other insects, laboratory-reared larvae have been shown to harbor significantly less diverse bacterial microbiomes than their wild counterparts [30], [31]. Laboratory strains of *D. melanogaster* have been reported to carry the bacterial genera *Lactobacillus*, *Acetobacter* and *Enterococcus* [21], [27], [28], [32]. Although these taxa are present in most studies, there is also a possible “lab effect” where different labs have different bacteria [28]. Many of the same bacterial genera (although not always the same species) were found in natural *D. melanogaster* populations in the eastern United States [32], [33]. However, given the worldwide distribution of *Drosophila* and the tremendous variation in *Drosophila* ecology, these taxa may represent only a small fraction of the bacterial communities associated with flies in the wild. A better knowledge of these communities is necessary to understand the role of symbiosis in *Drosophila* physiology, ecology, and evolution.

To explore the bacterial communities associated with this speciose and ecologically diverse lineage, and to identify the factors shaping these communities, we surveyed natural populations of 14 species of *Drosophila* and two closely related genera (*Scaptodrosophila* and *Microdrosophila*). Although we acknowledge that non-bacterial microbes such as archaea and yeasts are likely associated with these hosts, we focused our survey on the bacterial portion of the microbiome because of its known importance to animal and *Drosophila* biology. We shall use the term “bacterial microbiome” to refer to what was sampled in this study. We used culture-independent 16S ribosomal DNA (rDNA) polymerase chain reaction (PCR) amplification and sequencing to characterize the bacterial communities associated with each population. To sample the widest spectrum of fly-associated bacteria, collections were selected from as large a swath of *Drosophila* ecology, phylogeny, and geography as possible. Flies were collected directly from their natural feeding substrates including rotting fruit, flowers, mushrooms, and cacti, without the use of any artificial baits, from locations on both coasts of North America, Hawaii, Australia, Southeast Asia, and Seychelles, Africa. In addition to the natural survey, controlled laboratory experiments were performed to further determine the role of environment and host species in shaping the bacterial communities. This combined approach allows us to address several previously unexplored questions. Do the bacterial communities associated with *Drosophila* exhibit the same diversity as their hosts? What factors are most important in shaping the differences between symbiont communities of different host species? How does the composition and structure of these communities compare to the bacterial microbiomes of other taxa, particularly mammals? Finally, is the bacterial microbiome of lab strains used in experimental research representative of natural bacterial communities?

## Results

### Data summary

*Drosophila* samples were collected with the help of many colleagues around the world (see Acknowledgments, Table 1 and Dataset S1). Flies were either washed in sterile water (to remove cuticular bacterial cells) or were dissected to obtain just their crops and digestive tracts. After DNA extraction, rDNA PCR amplification was done with bacterial specific primers. The 16S rDNA amplicons were cloned, transformed, and Sanger sequenced from both ends. For 50% of the clones, the two reads did not overlap and therefore a concatenated read was made by inserting gap characters in the space between the two reads.

**Table 1:** Bacterial community samples used in this study.

Naturally Collected Flies			
Library Name	Species	Diet	Location
ELA	*D. elegans*	*Alpinia* Flowers	Hsinchu, Taiwan
ELD	*D. elegans*	*Brugmansia* Flowers	Hsinchu, Taiwan
FLV	*D. flavohirta*	*Syzigium* Flowers	NSW, Australia
FNS	*D. falleni*	Mushroom, *Russula sp*.	Stony Brook, NY
HCF	*D. hydei*	Citrus Fruit	Winters, Ca
HPM	*D. hydei*	Pomegranate Fruit	Winters, Ca
HPP	*D. hydei*	Opuntia Fruit	Arboretum, Davis, Ca
ICF	*D. immigrans*	Citrus Fruit	Winters, Ca
IMH	*D. sp. aff. immigrans*	*Hibiscus* flowers	Captain Cook, Hawaii
MAG	*D. sulfurigaster*	Mango Fruit	Waimanu, Hawaii
MAH	*D. melanogaster*	Grapes	Mahoney Winery, Napa, Ca
MAW	*D. melanogaster*	Grapes	Mahoney Winery, Napa, Ca
MIC	*Microdrosophila sp.*	Shelf Mushroom	Malaysia
MOV	*D. mojavensis + D. arizonae*	Agria Cactus	Sonora, Mexico
POM	Unidentified *Drosophila*	*Ipomoea* Flowers	Waimanu, Hawaii
PON	Unidentified *Drosophila*	Pandanus Fruit	Waimanu, Hawaii
SCA	*Scaptodrosophila hibiscii*	*Hibiscus* Flowers	Queensland, Australia
SEC	*D. sechellia*	*Morinda* Fruit	Seychelles, Africa
TBB	*D. melanogaster*	Citrus Fruit	Winters, Ca
TKM	*D. takahashii*	*Morinda* Fruit	Captain Cook, Hawaii

Laboratory Collected Flies			
Library Name	Species	Diet	Location
CAN	*D. melanogaster* Canton-S	Bloomington media	Kimbrell lab
ORF	*D. melanogaster* Oregon-R (Females)	Bloomington media	Kimbrell lab
ORM	*D. melanogaster* Oregon-R (Males)	Bloomington media	Kimbrell lab
WOB	*D. melanogaster* (WO) Bodies	Bloomington media	Kopp lab
WOE	*D. melanogaster* (WO) External Wash	Bloomington media	Kopp lab
WOG	*D. melanogaster* (WO) guts	Bloomington media	Kopp lab
WOL	*D. melanogaster* (WO) 3rd Instar Larvae	Bloomington media	Kopp lab
WOP	*D. melanogaster* (WO) Late Pupa	Bloomington media	Kopp lab
MED	Media Sample	Bloomington media	Kopp lab
Diet experiment (details in text)			
XDA	*D. melanogaster* (WO)	Agar	Kopp lab
XDE	*D. melanogaster* (WO)	EtOH	Kopp lab
XDM	*D. melanogaster* (WO)	Bloomington media (3 days)	Kopp lab
XDO	*D. melanogaster* (WO)	Bloomington media (Start)	Kopp lab
XDS	*D. melanogaster* (WO)	Sugar	Kopp lab
XDY	*D. melanogaster* (WO)	High Yeast	Kopp lab
Host species experiment (details in text)			
XYE	*D. elegans*	High Yeast	Kopp lab
XYM	*D. melanogaster* (WO)	High Yeast	Kopp lab
XYV	*D. virilis*	High Yeast	Kopp lab
XYX	External Wash All 3 species	High Yeast	Kopp lab

All 20 naturally collected samples were obtained without the use of artificial baits. All samples represent either externally washed whole bodies or dissected intestines, unless otherwise noted. All laboratory samples are from the University of California, Davis. Further details, including media composition, are provided in Dataset S1 and Text S1.

After all preliminary filtering, our dataset consisted of 3243 nearly full-length high quality sequences representing 39 host samples (which we refer to as libraries) (Table 1 and Dataset S1). This dataset excluded 421 clones that were only sequenced from one end, 65 sequences with fewer than 300 non-gap characters, 76 sequences that were identified as chimeric, 9 that appeared to be chimeric based on conflicting taxonomy assignments of the 3′ and 5′ reads, 3 chloroplast sequences, and 351 sequences of likely endosymbionts such as *Wolbachia* and *Spiroplasma* (which will be addressed in a separate section). Because small sample sizes can lead to inaccurate diversity measures [34], two libraries containing a total of 28 sequences were removed completely. The 39 remaining libraries vary in size from 26 to 223 sequences, with an average of 83.2 ± 37.4. Most libraries (29 of 39) contain between 63 and 97 sequences. 20 libraries containing 1850 total sequences are from wild-caught hosts, while the remaining libraries and sequences came from laboratory samples and experiments. Full tables containing each library's identifier, size, the host species from which it was collected, location and date of collection, and other information are given in Table 1 and Dataset S1.

Clustering with *mothur* [35] using the average neighbor algorithm with 0.03 cutoff (corresponding to 97% sequence similarity) creates 139 operational taxonomic units (OTUs), the largest of which contains 638 sequences. 66 OTUs are singletons (i.e., there is only a single sequence in the OTU) and 110 OTUs contain 10 or fewer sequences. The average OTU contains 23.3 sequences (standard deviation  = 78).

Phylogenetic analysis was performed using *FastTree* [36]. Included in this analysis (and many other comparisons throughout this study) were many previously identified *Drosophila*-associated bacteria [21], [32], [33], [37].

### Dominant bacterial taxa associated with *Drosophila*

**Figure 1:**
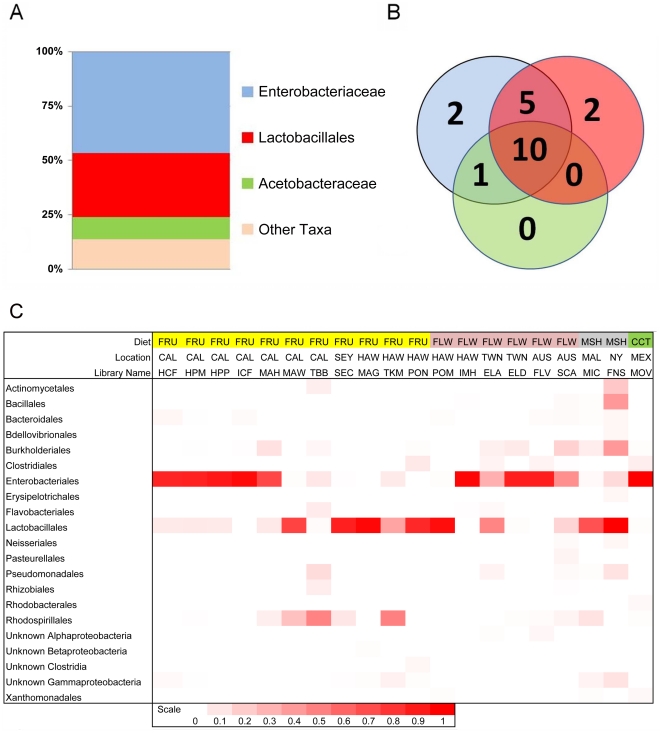
Composition and distribution of dominant bacterial taxa within 20 natural populations of *Drosophila*. A. Pooled samples across all species, diets and locations. “Other taxa” represents 34 families with an average abundance of <0.05% and 18 orders with an average coverage of <1%. B. Venn diagram representing the presence of these taxa within the 20 *Drosophila* populations. The numbers in the circles indicate how many populations contain at least one member of each of the three dominant bacterial taxa. Note that the Enterobacteriaceae and the Lactobacillales are almost universally found, each being found in 18 and 17 different populations, respectively. 10 populations contain all three dominant bacterial taxa. C. Relative abundance of bacterial orders within 20 wild *Drosophila* populations. Dark red indicates 100% of sample is composed of that order and white indicates 0% (exact scale at bottom). Note that each population is dominated by either the Enterobacteriales (all family Enterobacteriaceae), the Rhodospirillales (all family Acetobacteraceae), or the Lactobacillales. Diet Key: FRU = Fruit; FLW = Flower; MSH = Mushroom; CCT = Cactus. Location Key: CAL = Northern California; SEY = Seychelles; HAW = Hawaii; TWN = Taiwan; AUS = Australia; MAL = Malaysia; NY = New York; MEX = Mexico. Library identifiers are given in Table 1.

Four bacterial families representing three orders make up 90% of all sequences within our dataset. These include Enterobacteriales: Enterobacteriaceae (60%), Rhodospirillales: Acetobacteraceae (9%), and Lactobacillales: primarily Lactobacillaceae and Enterococcaceae (21%) (Figure 1A and Table 2). 14 other orders comprise the remaining 10% of the dataset. All wild populations are dominated by at least one of the three major clades, and many *Drosophila* species carry all three of them (Figure 1B and 1C). Although no core bacterial microbiome (a set of taxa present in *all* samples) emerges, Enterobacteriaceae and Lactobacillales come close, being found in 18 and 17 out of 20 wild *Drosophila* populations, respectively (Figure 1B). There is an interesting reciprocal relationship between these two taxa (Figure 1C). Each of the five host samples which lacks one of these groups is dominated (>84%) by the other one. In only two populations (ELA and SCA) do Lactobacillales and Enterobacteriaceae each make up at least 15% of the bacterial microbiome; both of these are flower-feeding flies with highly diverse bacterial microbiomes. In all the other samples, the abundance of the more dominant microbe is, on average, 44 times greater than the other one.

**Table 2:** Proportions of abundant genera within laboratory and wild-collected *Drosophila*.

Order/Family	Genus	Lab	Wild	Grand Total
Acetobacteraceae	*Acetobacter*	0.03	0.07	0.05
	*Commensalibacter*	0.03	0.03	0.03
Enterobacteriaceae	*Providencia*	0.29	0.01	0.13
	*Serratia*	0.07	0.04	0.05
	*Shigella*	0.03	0.06	0.05
	*Erwinia*	0.00	0.01	0.01
	*Pantoea*	0.00	0.05	0.03
	*Enterobacteriaceae Group Orbus*	0.39	0.29	0.20
	Other Enterobacteriaceae	0.01	0.01	0.01
Lactobacillales	*Enterococcus*	0.00	0.04	0.02
	*Lactobacillus*	0.11	0.06	0.08
	*Vagococcus*	0.00	0.16	0.09
	Other Lactobacillales	0.00	0.04	0.03
	All other Taxa	0.04	0.14	0.09
	Total Number of Libraries	19	20	39
	Total Number of Sequences	1393	1850	3243

Lab and wild columns represent all pooled samples. Genus names were assigned based upon the RDP classification, OTU membership, and phylogenetic placement relative to sequences from GenBank (see text for details).

#### Enterobacteriaceae

The Enterobacteriaceae, representing ∼60% (1956 out of 3243) of the sequences in our analysis, are a large family that includes many animal and plant associated bacteria. They are found as free living associates of many insects, including *Drosophila melanogaster* [32]. Several lineages are endosymbiotic and required for insect nutrition, defense from parasites, and tolerance of heat stress [38], [39], [40], [41]. Almost every wild and laboratory host contains some Enterobacteriaceae although it is notably absent from both distantly related mushroom-feeding species (Figure 1C).

1069 of our sequences, or nearly a third of the total dataset, form a closely related group within the Enterobacteriaceae (Figure S1). The closest type strains in the RDP database are within the family Pasteurellaceae, although the entire clade is nested within the Enterobacteriaceae. The closest named isolate is *Orbus hercynius gen. nov. sp. nov.,* which was isolated from the feces of a wild boar [42]. We have thus designated this entire lineage as “*Enterobacteriaceae Group Orbus”.* Although there is only one instance of members of this clade being previously found with *Drosophila* [33], it is highly abundant in both laboratory and wild *Drosophila* samples (548 and 521 sequences, respectively). Interestingly, the two *Enterobacteriaceae Group Orbus* OTUs with the largest number of sequences show a reciprocal distribution in the laboratory and wild host samples: one includes 539 out of 638 sequences in the lab, and the other 389 out of 392 sequences in the wild (Figure S1). In natural populations, representatives of these OTUs are not restricted to any single diet type and are found in fruit-, flower-, and cactus-feeding flies (Dataset S2). Many of the related sequences in Genbank were isolated from bee guts [43], [44], [45], [46]. Finally, despite that fact that 548 of the 1393 sequences isolated from laboratory samples belong to *Enterobacteriaceae Group Orbus*, no representatives have been found using standard culturing methods (data not shown).

Several of the other Enterobacteriaceae genera associated with *Drosophila* are closely related to opportunistic animal pathogens (Figure S2). Species in the genera *Providencia*, *Serratia*, and *Shigella* are common associates of the animal intestinal tract [47]. Several *Providencia* and *Serratia* species are used as model pathogens of *Drosophila*, as they elicit an immune response when introduced into the body cavity [48]. We find that *Providencia* is the most common genus found with laboratory flies and is present in 12 samples (Dataset S2). In contrast, *Serratia* is rare in the *Drosophila* intestine, but much more abundant on the exterior fly surface (Dataset S2). Flower-feeding flies, such as *D. elegans* and *D. flavohirta*, are unusual in having substantial internal *Serratia* communities. *Shigella* is less prevalent overall, and 67% of all *Shigella* sequences come from a single sample of wild-caught *D. melanogaster* (Library MAW; Dataset S2).

In addition to animal pathogens, several Enterobacteriaceae genera such as *Erwinia* and *Pantoea* contain plant pathogenic species. 109 sequences from 6 *Drosophila* samples are either *Erwinia* or *Pantoea* (Figure S2, Dataset S2). Interestingly, 98% of these sequences come from flower-feeding flies. *Pantoea* is present on both samples of *D. elegans*, and is represented by two distinct OTUs (Dataset S2).

#### Acetobacteraceae

The family Acetobacteraceae contains several genera collectively known as the acetic acid bacteria [49]. These obligate aerobic microbes thrive on high-energy substrates and are usually limited by nutrients other than their primary carbon source. They are common in sugary, acidic and alcoholic habitats, such as fruits and flowers. Possibly due to these habitat preferences, they are commonly associated with insects that consume sugar rich diets [50].

*Commensalibacter intestini*, originally isolated from *D. melanogaster* intestines, is a novel Acetobacteraceae species and genus proposed by Roh et al., 2008. In our data, an OTU of 97 sequences is identical to this cultured microbe (Figure S3; OTU TKM 092). This OTU is found in both wild and lab environments, primarily in fruit-feeding flies, but also in small amounts in mushroom feeders (Dataset S2). Most of the top BLAST hits in Genbank are from previous wild-caught *D. melanogaster* studies [32], [33]. In the *Acetobacter* genus, two OTUs represented by 153 and 15 sequences are found in both wild and laboratory environments, mainly in fruit-feeding flies (Dataset S2). The more abundant OTU is >99% identical to *A. malorum* isolated from *D. melanogaster* [21] (Figure S3; OTU TBB 298), and many of the top Genbank BLAST hits are from wild *D. melanogaster* [33]. The less abundant OTU is >99% identical to *A. pomorum* [21] (Figure S3; OTU MAW 008).

One apparent difference between our survey and previous *Drosophila* studies is the nearly complete lack of *Gluconobacter* and *Gluconacetobacter* within our samples. Four *Gluconobacter* sequences were found in *D. melanogaster* feeding on citrus fruit, and these are very closely related to those discovered in previous *Drosophila* studies (Figure S3, OTU TBB 129). *D. melanogaster* populations in the eastern US were found to contain a much higher diversity and abundance of *Gluconobacter* [33] (Figure S3). *Gluconacetobacter*, which was present in small numbers in those studies, is not found in our samples at all.

#### Lactobacillales

Lactobacillales (phylum Firmicutes) are widespread in the environment, and many are associated with animal hosts and fermenting plants. In our survey, Lactobacillales are represented mainly by the genus *Lactobacillus* (251 sequences) and the family Enterococcaceae (365 sequences). Of lesser abundance are the genera *Leuconostoc* and *Lactococcus*.

Lactobacilli are Gram-positive, acidophilic bacteria usually found on nutrient-rich resources. Several species, notably *Lactobacillus plantarum*, are routinely found within the mammalian digestive tract [51], [52]. We recovered two *Lactobacillus* OTUs that are >99% similar to cultured isolates of *L. plantarum* and *L. brevis* isolated from *D. melanogaster* in our lab (Figure S4; OTU WOG 027 and OTU MAW 097, respectively). These OTUs contained 171 and 68 sequences, respectively, and were found in both lab and wild samples, particularly in *D. melanogaster* collected on rotting grapes (Dataset S2).

*Enterococcus* is a very common inhabitant of insects, humans, and other mammals, possibly due to its tolerance of low pH environments and the ability to survive both hypotonic and hypertonic conditions [53]. In our survey, *Enterococcus* was found almost exclusively in wild-caught samples. An OTU containing 70 sequences was mainly found in a mushroom-feeding species of *Microdrosophila* (Library MIC, Dataset S2). This OTU is 97% identical to *E. faecalis*, a well-known commensal of mammals that is responsible for many hospital acquired infections in humans [53] (Figure S5; OTU MIC 001). A second Enterococcaceae clade of interest is sister to the genus *Vagococcus* and consists of 272 sequences from 3 OTUs (Figure S5; OTUs PON 059, SEC 085 and POM 057). The largest of these (210 sequences) is found almost exclusively in 3 samples of fruit-feeding flies. Interestingly, these samples came from two very distant sampling sites (Hawaii and Seychelles). Conversely, a very closely related OTU containing 55 sequences is found in only one sample (an unidentified Drosophilid from *Ipomoea* flowers in Taiwan) (Dataset S2 and Figure S5; OTU POM 057). Several sequences in GenBank closely related to both of these OTUs were isolated from larvae of humus-feeding beetles [54].

#### Candidate endosymbionts

The order Enterobacteriaceae contains many obligate endosymbiotic bacteria including *Buchnera* (in aphids), *Wigglesworthia* (in tsetse flies) and *Baumannia* (in sharpshooters) [8], [38], [55]. None of the sequences in our survey fall within the monophyletic clade comprised of these endosymbionts. Similarly, no close relatives of the facultative defensive symbionts such as *Regiella insecticola* and *Hamiltonella defensa* [41] were found. This does not preclude the possibility that novel endosymbionts are present in the surveyed species, since our methods do not allow them to be distinguished from free-living bacteria.

We did observe two well-known *Drosophila* endosymbiotic bacteria, *Wolbachia* and *Spiroplasma*. 317 *Wolbachia* sequences were seen in 9 libraries (3). *D. melanogaster* in Northern California were particularly infected with *Wolbachia*, whereas other populations had much lower infection loads (Dataset S3). OTU analysis places these *Wolbachia* into two distinct clusters. 312 sequences are >99% similar to each other and contain all the sequences from the Northern California populations, as well as 23 sequences from *D. takahashii* from Hawaii. A BLAST search confirms this strain as being closely related to *Wolbachia pipiens* from *Drosophila simulans* at 98% identity [56]. 5 additional *Wolbachia* sequences came from *Scaptodrosophila* (Australia) and *D. sechellia* (Seychelles, Africa) (Dataset S3). We also identified 26 *Spiroplasma* sequences (class Mollicutes, phylum Tenericutes). A single sequence from *D. hydei* is closely related to other *Spiroplasma* strains from *D. hydei* [57]. The remaining 25 sequences all originate in *D. takahashii,* and are similar to a group of male-killing *Spiroplasma* found in a variety of insects. Several *Drosophila* species, including *D. ananassae* and *D. atriplex*, are infected with closely related *Spiroplasma* [58].

#### Other bacterial taxa

In addition to the four dominant families, 31 additional families representing 18 different orders are associated with *Drosophila* populations. Nearly all of these are present in amounts of less than 1% of the total bacterial microbiome (Dataset S2). Several of these taxa are known symbionts of animals [12]. For example, the Clostridiales, the Bacteroidales, and the Actinomycetales each make up ∼1% of the *Drosophila* bacterial microbiome. The most widespread of these taxa, the Bacteroidales genus *Dysgonomonas*, is present in 8 separate *Drosophila* populations and is not restricted to any one locality, species, or diet type (Dataset S2).

### Diversity of bacterial communities

**Figure 2:**
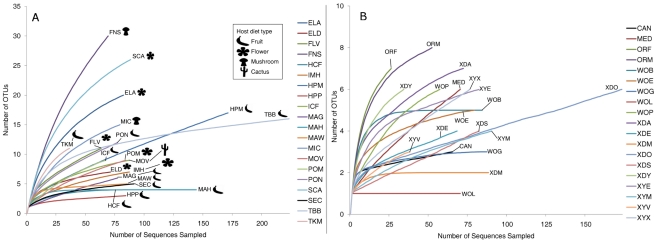
Rarefaction analysis of observed richness within *Drosophila*. All calculations were performed using *mothur* [35]. OTUs were defined at the 3% divergence threshold using the average neighbor clustering algorithm. Library identifiers are given in Table 1. Note the different scales of the Y-axis in panels A and B. A. Rarefaction analysis of wild populations of *Drosophila.* B. Rarefaction analysis of laboratory collected samples.

OTU richness, evenness, and overall diversity vary widely among host samples (Table S1). As many as 30 OTUs were present in some samples such as *D. falleni* collected on *Russula* mushrooms, while five or fewer OTUs were found in 5 different samples. For example, *D. hydei* collected from either citrus fruit or prickly pear are found with four or less bacterial OTUs, and a single Enterobacteriaceae OTU represents at least 85% of each of these bacterial microbiomes. Similarly, *D. sechellia* collected on *Morinda* fruit is dominated by a single *Lactobacillales* OTU (84%), leading to very low bacterial community richness and evenness (Dataset S2). Rarefaction analysis, which helps determine how close the sampling effort came to fully describing the community, shows that different host communities differ greatly in richness and were sampled at different depths (Figure 2A). The least diverse samples are those collected from fruit-feeding hosts, while the flower- and mushroom-feeders tend to have more diverse bacterial communities. For the communities that have not been sampled to completion, the situation exists in which rare, and potentially important, taxa have not been identified.

Community similarity (beta-diversity) between samples was calculated for each of the 190 comparisons between the 20 wild populations (Dataset S4). In 27% of these comparisons, no OTUs are shared between the two samples. The two *Drosophila* that share the highest proportion of their bacterial microbiomes are *D. hydei* collected from citrus fruit and prickly pear fruit (samples HCF and HPP, respectively, Dataset S4).

In contrast to the bacterial communities associated with wild populations, laboratory samples are much less diverse and so were sampled nearly to completion (Figure 2B). Chao1 analysis [59] predicts an average of 6.3 OTUs per sample, and most libraries have >80% coverage (Table S2). It is interesting to note that some of the most OTU-rich communities are present on the culture media and on the external surfaces of flies (MED and XYX) (Table S2). This suggests that flies are able to exclude many of the external microorganisms present on the feeding substrate, allowing only a subset to persist in their digestive tract.

In both wild and lab host samples, most of the bacterial diversity is found at short phylogenetic distances, since most samples share the same dominant orders and families (Figure 1). This distribution produces a typical “hockey stick” pattern found in many animal-associated microbial communities (Figure S6) [60].

### Differences between *Drosophila* and mammalian bacterial communities

**Figure 3:**
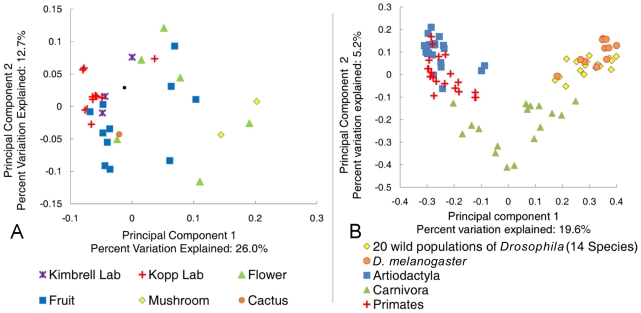
Principle component analysis of the natural *Drosophila* microbiome. All sequences were aligned and trimmed as described in the text. A single rooted tree for each PC analysis was generated using *FastTree* [36]. PC analysis was done with the *FastUniFrac* web application [62]. A: Comparison of the *Drosophila* microbiome with respect to diet type. All 20 naturally collected samples are included along with the laboratory samples from adult *Drosophila* feeding on rich Bloomington media (Text S1). B: Comparison of the natural *Drosophila* bacterial microbiome and the mammalian bacterial microbiome. *D. melanogaster* data is from Corby-Harris et al., 2007 [33]. Selected mammalian orders are from Ley et al., 2008a [12].

To put the *Drosophila* bacterial microbiome in perspective, we compared the 20 wild-caught samples to published mammalian datasets [12] and previous studies of naturally isolated *D. melanogaster* [33]. These studies are well suited for effective comparison to our data because they use culture-independent, long-read Sanger sequencing that allows closely related OTUs to be resolved, and because they represent a large taxonomic breadth and/or include many samples from a wide geographic area. Principal component analysis (PCA) shows that the *Drosophila* bacterial microbiome from our study is similar to previous *D. melanogaster* samples, but is clearly distinct from the microbiome found in the mammalian orders Artiodactyla, Carnivora, and Primates (Figure 3B). Despite the relatively tight clustering of *Drosophila* samples, some differences between separate studies are apparent (Table S3). Notably, the Enterobacteriaceae, which are the dominant taxon in our global survey, are almost absent from two previous *Drosophila* studies [21], [33]. Although Enterobacteriaceae comprise a large proportion of the bacterial microbiome within a single Massachusetts population [32], the dominant genera in that sample were *Enterobacter* and *Klebsiella*, which are not present in our survey. The high abundance of Acetobacteraceae in the Massachusetts population may be caused by the fruit bait used during sample collection in that study [32].

The dominant bacterial order in all three mammalian orders is the strictly anaerobic Clostridiales, which is rarely found in *Drosophila* (Table S4). The Enterobacteriaceae are not found or are minimal residents of the Artiodactyla and Primate guts, respectively. While this family is present in high amounts within the Carnivora, the dominant genera, *Escherichia* and *Shigella*, are not common in flies (Dataset S2). A similar pattern is found for the Lactobacillales. This order is found in relatively high numbers in the Carnivora and Primates (20% and 9% respectively) [12], but the major genus in mammals (*Streptococcus*) is found at less than 0.5% abundance in wild flies (Dataset S2). Finally, Acetobacteraceae are not present in any of the three mammalian orders [12]. The only bacterial genus present in appreciable numbers in both mammals and *Drosophila* is *Lactobacillus*. This genus is found in Artiodactyla (2%), Carnivora (3%), Primates (2%), and *Drosophila* (3%) [12] (Dataset S2).

Flies also differ from mammals in the overall patterns of bacterial microbiome diversity. The richness of *Drosophila* bacterial communities is dramatically lower than in mammals, although community evenness is comparable (Table 3). Additionally, we find that many OTUs are present in taxonomically and ecologically diverse *Drosophila* populations (Dataset S2) and that the proportion of bacterial OTUs that are unique to a single host sample is consistently lower in *Drosophila* than in mammals (Figure S7).

**Table 3:** Average diversity measurements for *Drosophila* and mammals.

		Global Survey	Laboratory *Drosophila*	*D. melanogaster*	Artiodactyla	Carnivora	Primates
Chao1 Richness	Average	17.13	5.86	19.06	542.68	87.31	307.20
	SD	14.75	2.66	11.52	360.89	56.47	133.76
Shannon Diversity	Average	1.38	0.84	2.03	3.94	2.18	3.59
	SD	0.77	0.52	0.51	1.08	1.10	0.73
Shannon evenness	Average	0.58	0.54	0.88	0.87	0.62	0.80
	SD	0.20	0.24	0.05	0.20	0.25	0.12

All calculations were performed using *mothur* [35]. OTUs were defined at the 3% divergence threshold using the average neighbor clustering algorithm. *D. melanogaster* data is from Corby-Harris et al., 2007 [33]. Selected mammalian orders are from Ley et al., 2008a [12]. Details regarding calculations can be found at http://www. mothur.org/wiki/Calculators.

### Effect of host diet on the composition of natural bacterial communities

**Figure 4:**
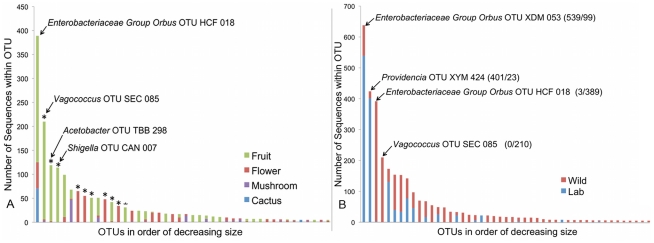
Composition of OTUs. A. Composition of OTUs within naturally collected flies with respect to diet type. Asterisks indicate OTUs which derive more than 95% of their sequences from a single diet type. OTU names for the four largest OTUs are given. OTUs with fewer than 5 sequences are omitted. B: Composition of all OTUs with respect to sampling environment (*i.e*. laboratory or wild environment). OTU names and the absolute number of sequences from lab and wild populations, respectively, are given for the four largest OTUs. OTUs with fewer than 5 sequences are omitted.

To estimate the role of host diet in shaping bacterial microbiome composition, we compared taxonomically diverse *Drosophila* species collected from different types of food sources. Our survey contains 11 samples of fruit-feeding flies and 6 samples of flower-feeders. *UniFrac* analysis [61], [62] shows that flies subsisting on these two diets have significantly different bacterial microbiomes (p<0.01). One major difference is the absence of Acetobacteraceae in flower-feeding flies (Table 4). This may be due to the fact that Acetobacteraceae can thrive under the low pH and high ethanol conditions present in fermenting fruit. The same argument can be made for *Lactobacillus*, an acidophilic genus associated with high resource habitats, which is present at higher abundance in fruit-feeding flies (Table 4). In contrast, the genera *Serratia* and *Pantoea* (Enterobacteriaceae) are found in much higher proportions in flower-feeders (Table 4). Many of the largest OTUs are found only, or mainly, in association with one diet type (Figure 4A). Of the 14 largest OTUs, which contain 75% of all sequences, 10 derive >95% of their sequences from a single diet type (Figure 4A). In general, the difference between fruit- and flower-feeders is consistent and can be attributed to multiple host samples within each category. An exception to this pattern is *Shigella*, whose apparent abundance in fruit-feeding flies is due almost entirely to a single library (*D. melanogaster* from rotting grapes, Sample MAH) (Dataset S2).

**Table 4:** Taxonomic differences between the bacterial microbiomes of flower and fruit feeding *Drosophila*.

Order/Family	Genus	Flower	Fruit
Acetobacteraceae	*Acetobacter*	0.00	0.11
	*Commensalibacter*	0.01	0.03
Enterobacteriaceae	*Enterobacteriaceae Group Orbus*	0.20	0.31
	*Pantoea*	0.19	0.00
	*Serratia*	0.14	0.01
	*Shigella*	0.00	0.10
Lactobacillales	*Lactobacillus*	0.01	0.08
	*Vagococcus*	0.13	0.18
	Other Taxa	0.32	0.18
	Total Number of Sequences	458	1160
	Number of Populations Sampled	6	11

Each column represents the pooled results from all flies obtained from each diet type. Diet details are found in Table 1.

Similarities among the bacterial communities of wild populations and laboratory strains were summarized with PCA using *UniFrac* (Figure 3A). We find that the majority of fruit feeding flies occupy a distinct region within PC space, while the two mushroom feeders are mostly separated from the other samples. In congruence with the taxonomic similarity between the cactus feeding population and the fruit feeders, we find that the *D. mojavensis* sample clusters near the fruit associated flies.

Some differences are also apparent within diet types. In particular, *D. elegans* was collected simultaneously from *Alpinia* and *Brugmansia* flowers (Samples ELA and ELD). These collections were made less than 10 meters apart and almost certainly represent a single fly population. Therefore, any differences in their bacterial communities are most likely due to the different food sources. We find that *D. elegans* collected on *Alpinia* has a much higher amount of Leuconostocaceae and Streptococcaceae (phylum Firmicutes), while those collected on *Brugmansia* are dominated by Enterobacteriaceae (phylum Proteobacteria) (Dataset S2). *Alpinia*-collected flies also show much higher bacterial microbiome diversity than *Brugmansia*-collected flies (Chao1  = 23 vs 7.5) (Table S1). Although it is possible that individual flies travel between host plants, these switches are clearly insufficient to overcome the effect of diet.

Both mushroom-feeding populations were associated with a high amount of Lactobacillales, specifically *D. falleni* had 30% *Vagococcus* and *Microdrosophila sp*. had 57% Enterococcus (Dataset S2). *D. falleni* is also notable because its bacterial microbiome contains 16% each of both Bacillales and Burkholderiales, two orders that are otherwise rare in *Drosophila* bacterial microbiomes (Figure 1C). The mushroom-feeding species are also marked by relatively high community richness and diversity, especially compared to fruit-feeding *Drosophila* (Table S1 and Figure 2A). The single cactus-associated population is very similar to many fruit feeders both in composition (84% *Enterobacteriaceae Group Orbus*) (Dataset S2) and diversity (Table S1).

### The bacterial microbiome of laboratory flies has reduced diversity and distinct composition

A major benefit of the *Drosophila* model is the experimental flexibility it provides in a laboratory setting. However, OTU classification and rarefaction analysis show that lab-raised flies contain a much lower richness and diversity of bacteria compared to wild-caught flies (Figure 2, Table 3, Table S1 and Table S2). At the broadest level, the wild and laboratory samples are similar in that both are composed mainly of Enterobacteriaceae, Lactobacillales, and Acetobacteraceae (Table 2). However, 90 of the 139 total OTUs are present only in wild samples, while six are found only in lab samples (Figure 4B). Most of these OTUs are rare, so that the majority of sequences in our survey belong to OTUs that are found in both wild and lab hosts (Figure 4B). The four largest OTUs, which together comprise over half of the entire dataset, are composed of both wild and laboratory sequences. It should be noted, however, that each of these four OTUs is composed primarily (>95%) of either wild or laboratory sequences (Figure 4B).

PCA (Figure 3A) further emphasizes the reduced diversity and distinct composition of the bacterial microbiome of laboratory flies. We find the laboratory populations in a subset of the total PC space occupied by the wild populations. Specifically, the laboratory samples' PC space is near that of the fruit feeding *Drosophila*, which could be explained by the nutritional similarity of these sugar rich diets.

Many of the bacterial strains found in this study are closely related to those from previous laboratory studies of *Drosophila*. Five strains that are common in our lab samples (*Acetobacter malorum, A. pomorum, Commensalibacter intestini, Lactobacillus brevis*, and *L. plantarum*) are >99% identical to previously indentified cultured isolates of *D. melanogaster* [*21*] (Figure S3 and Figure S4). A notable difference between our results and another previous study is that *Enterococcus* is virtually absent in our lab samples (Table S3), but comprises nearly 50% of the laboratory bacterial microbiome in that study [32].

### Experimental analysis of host species and diet effects on the intestinal bacterial microbiome

Our survey of natural bacterial communities suggests that host diet may be an important determinant of bacterial microbiome composition. We tested this hypothesis using laboratory experiments where diet and rearing conditions were carefully controlled. Starting with a large pool of isogenic *D. melanogaster*, we transferred 25 flies each to a different sterile diet and examined the resulting changes in their gut bacterial communities. We find that the high yeast diet, which is most similar in composition to our standard lab media, induced a similar bacterial microbiome with a high abundance of *Enterobacteriaceae Group Orbus* (Table 5). In contrast, the high ethanol and sugar-only diets resulted in a bacterial microbiome dominated by *Providencia*. Flies on the no-nutrient (agar-water) diet contained appreciable levels of both of these groups, but a quarter of their bacterial microbiome was composed of *Commensalibacter intestini* (Table 5). Flies kept on standard lab media showed little change in their bacterial microbiome after three days, suggesting that diet has a consistent effect on the bacterial microbiome. *UniFrac* analysis confirms a significant overall effect of diet in this experiment (p<0.01).

**Table 5:** Gut bacterial microbiome composition on different diets.

	Lab Media (Start)	Lab Media (3 days)	Sugar	Agar	EtOH	Yeast
*Providencia*	0.17	0.10	0.98	0.22	0.85	0.24
*Commensalibacter*	0.00	0.00	0.01	0.24	0.00	0.09
*Enterobacteriaceae Group Orbus*	0.82	0.90	0.01	0.50	0.13	0.65
Other Taxa	0.02	0.00	0.00	0.04	0.01	0.03
Total Number of Sequences	173	88	82	72	68	34

In a reciprocal experiment, we tested whether different host species develop different bacterial microbiomes when feeding on the same diet. Three distantly related Drosophilids that feed on different food sources in the wild, *D. melanogaster* (fruits), *D. elegans* (flowers), and *D. virilis* (sap fluxes and cambium), were reared together on the same media. We found that all three species had similar bacterial microbiomes at the end of this experiment (Table 6). The digestive tracts of each species contained between 72% and 94% *Providencia*. *UniFrac* analysis does not show significant differences between host species (p = 0.54). However, some differences between these species could be masked because the strains used in this experiment have been adapting to the laboratory environment for many generations. Additionally, laboratory *Drosophila* are likely exposed to a lower overall diversity of possible symbionts than their wild counterparts, further masking possible differences between host species.

**Table 6:** Gut bacterial microbiome composition in different species co-cultured on the same media.

	*D. elegans*	*D. melanogaster*	*D. virilis*
*Enterobacteriaceae Group Orbus*	0.10	0.01	0.00
*Lactobacillus*	0.07	0.01	0.00
*Providencia*	0.72	0.94	0.89
*Serratia*	0.09	0.03	0.05
Other Taxa	0.02	0.00	0.05
Total Number of Sequences	82	90	38

Our study spanned two years and used flies from two different labs at UC-Davis. The Kimbrell and Kopp lab flies had significantly different bacterial microbiomes, despite obtaining the same type of media from the same kitchen (p<0.01). The three dominant taxa in the Kopp lab are *Enterobacteriaceae Group Orbus*, *Providencia*, and *Lactobacillus*, while all three are at minimal amounts within the Kimbrell lab (all three combined equal 9% of the bacterial microbiome within *Drosophila* from the Kimbrell lab) (Table S5). Conversely, the dominant taxa in the Kimbrell lab are *Shigella* and *Variovorax*, which are not present in the Kopp lab. Even within the Kopp lab, the bacterial microbiome was different in experiments performed at different times (p<0.01) (Table S6). We propose that these inter- and intra-lab differences are the result of different sets of environmental communities that inhabit the labs and inoculate the fly stocks during routine maintenance. These observations suggest that some of the conflicting phenotypic results reported by different labs [27], [28] may be the result of different bacterial communities.

### Intestinal bacterial microbiome differs from the environmental bacterial community

The bacterial microbiome of *Drosophila* is likely environmentally acquired since, with the exception of *Wolbachia* and *Spiroplasma*, no evidence exists that bacterial communities are transmitted vertically within the egg. To ask whether the gut bacterial microbiome differs from the external bacterial community, we examined external washes of adults and their culture media (Table S7). *UniFrac* analysis shows a significant difference (p<0.01) between the external and internal samples of *D. melanogaster* grown on unsterilized media. Larvae also differ significantly from the media they feed on (p<0.01). The larval bacterial microbiome consisted entirely of *Enterobacteriaceae Group Orbus*, while the media also contained *Serratia*, *Providencia,* and *Lactobacillus*.

## Discussion

### *Drosophila* has a taxonomically restricted bacterial microbiome

Natural *Drosophila* populations have a remarkably restricted bacterial microbiome. Despite the phylogenetic, ecological, and geographical diversity of the hosts we surveyed, only a few bacterial clades are associated with all these flies. The families Enterobacteriaceae and Acetobacteraceae and the order Lactobacillales make up over 85% of natural *Drosophila* bacterial microbiome (Figure 1A). All *Drosophila* populations are dominated by at least one of these clades, and many host isolates have all three of them (Figure 1B). Although we find no strict core bacterial microbiome, Enterobacteriaceae and Lactobacillales are found in 18 and 17 of the 20 wild *Drosophila* populations, respectively. Each of the five samples that lack either of these groups is dominated by the other, and the two groups generally show a pattern of reciprocal abundance (Figure 1C). One possible explanation is that competitive interactions between the two groups allow only one of them to persist at a detectable level within the host digestive tract.

These three bacterial taxa are emerging as common microbial associates of insects. The Acetobacteraceae (*Acetobacter sp.*) have been found with bees, olive fruit flies, parasitic wasps and mealybugs [44], [63], [64], [65], [66]. Likewise, the Lactobacillales (such as *Lactobacillus*) are common symbionts of insects, notably bees and beetles [43], [65], [67], [68]. Finally, the most common Enterobacteriaceae found with *Drosophila* (*Enterobacteriaceae Group Orbus*) has found with numerous insect species, but especially bees (Figure S2) [43], [44], [45], [46], [69], [70], [71], [72].

This taxonomically restricted bacterial microbiome leads to interesting patterns of bacterial diversity. Many samples have very low observed and expected (Chao1) species richness (Table S1). These results stand in contrast with the highly diverse bacterial communities found in mammals [12] (Table 3). There is an important difference in sampling procedures: the mammalian samples each come from a single individual [12], while the *Drosophila* samples were isolated from multiple individuals. However, this difference would be expected to bias the results in the *opposite* direction, since different individuals are likely to carry slightly different bacterial communities.

Our laboratory studies show that the intestinal bacterial microbiome represents only a subset of the external bacterial communities (Table S7). This suggests that although the gut bacterial microbiome is environmentally acquired, the host exerts significant control over its composition. Since most environmental samples are composed of many phyla and are rarely dominated by just one or two lineages [73], we suggest that the low-diversity communities of *Drosophila* reflect the effects of strong host filtering. Whether this filtering is an adaptive function of the immune system or simply a by-product of the physiological conditions in the gut remains to be determined, but host control has previously been demonstrated in genetic experiments [21], [26]. The importance of bacterial microbiome restriction for host fitness is yet to be investigated, as well.

### The same bacterial lineages are associated with different host species, diets, and locations

Analysis of OTU-level data shows that individual OTUs are not specific to a single host species, diet type, or location, but are typically associated with many *Drosophila* populations. Although most OTUs (91 out of 127) present in wild flies are each found in one host sample, all these OTUs represent only a small percentage of the total fly bacterial microbiome (16%). Conversely, the dominant OTUs from each host population are usually found in other populations as well. In fact, we find that the most common OTU in 19 out of 20 populations is also found in other, often geographically distant, hosts. Several particularly wide-ranging OTUs are found in nearly half of all populations. In comparison with mammalian bacterial microbiomes [12], the fraction of OTUs unique to a single host sample is much lower (Figure S7).

The closest relatives of many bacterial lineages found in our survey were also detected in previous studies of *D. melanogaster*. For several common taxa (*Commensalibacter, A. malorum, A. pomorum, L. plantarum, and L. brevis*), the closest sequences in GenBank were isolated from *D. melanogaster*. Since few *Drosophila*-associated 16S sequences are available in GenBank, compared to the much greater number of non-host associated and mammalian-associated sequences, these similarities imply a pervasive association of these lineages with *Drosophila*. Overall, these patterns suggest that the bacteria associated with *Drosophila* display some level of host specificity. Since far-flung, ecologically diverse flies are associated with a common set of bacteria, “*Drosophila*” can be considered a selective environment that allows only certain taxa to persist.

### Host diet has a greater effect on the bacterial microbiome than host species

Previous studies have shown that the mammal-associated bacterial microbiome is shaped by both host phylogeny and host diet, while sampling location has little or no effect on community composition [12], [74]. Diet has also been shown to influence the bacterial composition of gypsy moth [30] and cotton bollworm [31] larval midguts. We find that host diet plays a substantial role in shaping bacterial microbiome composition in *Drosophila*, as well. This conclusion is supported both by the survey of natural communities and by controlled laboratory experiments. Although we were unable to quantify the role of host species in natural populations because many species were only represented by a single collection, laboratory populations of multiple co-habitating species showed no significant differences between their bacterial microbiomes.

These results suggest two possible hypotheses regarding the assembly of *Drosophila*–associated bacterial communities. One possibility is that the guts of different host species inhabiting the same food source provide suitable environments for the same bacteria. These bacteria could provide specific benefits to their hosts on that diet, so that phylogenetically distant *Drosophila* species evolve to allow the persistence of the same, diet-specific, bacteria. Alternatively, different substrates may harbor different bacterial communities and environmental acquisition of these bacteria may simply overwhelm any potential control by the host. As these hypotheses suggest different roles for the host (adaptive *vs.* passive), future experiments should take care to sample the bacterial community of the environment the host is interacting with.

If environmental acquisition is indeed the most important factor determining *Drosophila* bacterial microbiome composition, then two general observations are expected. First, patterns of host and symbiont co-speciation seen in closely related insect and mammalian groups should not be observed within *Drosophila* [17], [74]. Second, the genetic complementarily commonly found in tightly associated symbionts should be harder to evolve [8].

### Lab-raised flies are a limited model of natural host–microbe interactions

*Drosophila* has recently emerged as a powerful model for studying non-pathogenic host-microbe interactions. Several important genes that control host interactions with commensal intestinal bacteria have been identified, including *caudal* and *PIMS* (Lhocine et al., 2008; Ryu et al., 2008). Another study has shown that *Lactobacillus plantarum* can affect mating preferences (Sharon et al., 2010). Cox and Gilmore, 2007, have suggested that *D. melanogaster* is naturally colonized by the commensal/opportunistic pathogen *Enterococcus faecalis*, and can serve as a good model for *E. faecalis* pathogenesis. In all these studies, laboratory experiments serve as a proxy for the natural ecology of *Drosophila*-microbe interactions. However, in order to serve as an ideal model system, the lab bacterial microbiome should be a subset of the wild bacterial microbiome, and the most common wild taxa should be found in the lab.

We find that these conditions are only partially satisfied. The putative commensal bacterial genera studied by Ryu et al., 2008 are members of the family Acetobacteraceae (*Acetobacter, Glucoacetobacter, Commensalibacter*) and the genus *Lactobacillus* (*L. plantarum* and *L. brevis*). Ren et al., 2007 also identified *Acetobacter* and *Lactobacillus* as commensal bacteria in laboratory-reared flies. While all of these bacteria are present in some *Drosophila* populations, their abundance in wild samples is low and none are ubiquitous. In *D. melanogaster* samples *L. plantarum* and *L. brevis* comprise 7.7% and 9.7% of the total bacterial microbiome, respectively, whereas *Enterococcus*, *Commensalibacter* and *Glucoacetobacter* are not found at all. Only *L. plantarum* is found in all wild *D. melanogaster* samples.

*Drosophila* has been used for decades as a model for pathogenic bacterial infections. In some cases, it was applied to study bacteria that pose important threats to human health, such as *Bacillus anthracis* [*7*5], *Vibrio cholerae* [76], [77], *Salmonella typhimurium* [78]–[80], *Pseudomonas aeuruginosa* [81]–[86] and *Burkholderia cepacia* [78]. Other studies focused on elucidating the molecular mechanisms of fly immunity using known or suspected entomopathogens or phytopathogens, including species of *Serratia* [86], [87], *Erwinia* [88], [89], *Micrococcus* [90], and *Pseudomonas* [91], [92]. We find that, collectively, the above 8 microbes make up less than 10% of the total *Drosophila* microbiome, and none constitutes more than 3.5% individually. This indicates that they are relatively rare in wild *Drosophila* populations on the whole, although we cannot rule out the existence of some unsampled, heavily infected individuals.

While most of the well-studied lab bacteria are rare in natural populations, the reciprocal is also true – the most common bacteria in wild populations are not the most abundant *Drosophila* associates in the lab (Figure 4B), and have not been used as model bacteria in laboratory studies. A single group, *Enterobacteriaceae Group Orbus*, represents over 21% of all bacteria present with natural *Drosophila* populations and is nearly twice as abundant as the next most common genus. This clade is present in over half of all *Drosophila* populations, but has not been used in any laboratory studies. The second most common bacterium in wild *Drosophila*, a strain of *Vagococcus* (15% of total bacterial microbiome, present in 9 populations), has also never been used in *Drosophila* host-microbe studies.

One final consideration for laboratory studies concerns the lab- and time-dependent variation in bacterial communities. It has been previously suggested that discrepancies between reported phenotypes may be due to different bacterial communities present in different labs [28]. Indeed, we find that different laboratories at UC-Davis are home to completely different bacterial communities despite using the same media (Table S5). Even when genus-level taxonomies agree (as in *Serratia*), OTU clustering shows that different strains are present in different laboratories. Moreover, we find that bacterial community composition can change in the same lab over time (Table S6).

Despite these caveats, laboratory strains of *Drosophila* can still serve as a useful model of host-microbe interactions. For example, conclusions from the natural survey mesh well with laboratory experiments in highlighting the importance of diet in shaping the bacterial microbiome. We suggest that many experimental projects would benefit from determining and monitoring the composition of bacterial communities associated with fly strains. Awareness of this important aspect of host biology will lead to a better understanding of *Drosophila* physiology, ecology, and evolution.

### A model of *Drosophila* microbiome assembly

Our results suggest a model where the composition of gut bacterial communities is determined by three separate factors: diet, host physiology, and chance. Since all gut bacteria must first be ingested, bacterial taxa that thrive on the feeding substrates of the host species will have the greatest chance of colonizing the gut. The aerobic, and often high-nutrient environments frequented by *Drosophila* may present taxonomically and geographically distant fly populations with similar “source” bacterial communities. Furthermore, the quantitative differences between *Drosophila* feeding upon different food sources may be the result of exposure to different diet-specific bacterial communities. Next, within the range of microbes presented by the diet, some properties of the *Drosophila* intestinal environment determine which bacteria are allowed to persist. These properties may reflect conserved features of the *Drosophila* immune system as well as the physico-chemical conditions in the gut lumen – such as pH or the simple fact that, unlike the mammalian digestive tract, the *Drosophila* gut is most likely an aerobic environment. This may explain why the closest relatives of the dominant OTUs in our survey come from other insects, and why bacteria commonly associated with flies are very rare in diverse mammalian species and vice versa. At this time, it is not clear whether genetic variation between or within species can further bias the acquisition of symbionts. Although we do not detect an effect of host species in our study, it is possible that deeper sequencing will uncover quantitative effects of the host genotype, especially under controlled environmental conditions. Finally, within the boundaries set by the host diet and subject to host filtering, the microbiome of each population is likely determined by chance environmental encounters between flies and bacteria. This factor may explain both the lab effect and the change in bacterial communities over time observed in our lab samples. In the simplest scenario, each individual host would collect a random sample of permissible bacteria available in its environment. A further level of complexity may be added if one considers the interactions between bacterial taxa or their order of colonization. The reciprocal dominance of Enterobacteriaceae and Lactobacillales in *Drosophila* samples suggests that one or both of these processes may be important.

This model of microbiome assembly, while consistent with all our data, remains to be tested by more systematic environmental sampling and experimental analyses. It is also unclear whether it applies to other *Drosophila*-associated microbes such as yeast. Repeated sampling of multiple co-occurring species from the same feeding sources, analysis of individual variation in natural populations and laboratory settings, and characterization of bacterial communities native to the diet of each population will all be necessary to determine the relative importance of source bacterial communities, host control, and the vagaries of chance in shaping the gut microbiome.

The gut bacterial communities of *Drosophila* are likely to represent the most common type of animal microbiomes, where symbionts are free-living and horizontally transmitted and the host-symbiont associations are flexible and facultative. If this model is confirmed by future work, it may serve as a paradigm for the assembly of other animal microbiomes in nature. This framework may help us understand both the ecology of host-symbiont interactions and the functional impact of these interactions on the host.

## Materials and Methods

### Fly collection, dissection, and DNA extraction

*Drosophila* samples were collected with the help of many colleagues around the world (see Acknowledgments, Table 1 and Dataset S2). All samples were obtained from naturally occurring substrates and no artificial baits were used to attract flies. For collections done in Northern California, adults were immediately transferred to sterile no-nutrient media (agar-water) and transported to UC-Davis for dissection, which occurred within 2 hours of collection. For more remote field collections, flies were stored in 100% ethanol for transport.

Freshly collected flies were washed twice in 2.5% bleach and twice in sterile water. The entire gut was dissected in sterile insect saline and placed in sterile TES buffer (10 mM Tris-HCl [pH = 7,5], 1 mM EDTA, 100 mM NaCl). For flies stored in ethanol, dissection was not feasible because weakening of the fly tissues caused the gut to fragment. For these samples, the entire fly body was used after three washes with sterile water. To ensure adequate removal of external bacteria, each final wash was confirmed to be free of bacterial cells by PCR with universal bacterial primers and by plating onto rich media. In no case did the final wash show evidence of bacterial contamination. For a single sample (*D. melanogaster* reared in the Kopp laboratory), the first wash was saved for DNA extraction to characterize the external bacterial community. Seven to 20 fly bodies or guts were combined for most samples. In one exception (*D. melanogaster* bodies collected from rotting grapes, sample MAW) only a single body was used. On a single occasion, the bacterial community of laboratory media within the Kopp laboratory was sampled using 1 ml of media that had been inhabited by *D. melanogaster* for 7–10 days. Further details regarding sample collection dates, locations, and contents can be found in Dataset S1.

DNA was extracted from samples using a modification of the Bead Beater protocol [93]. The tissue was homogenized by grinding and three freeze/thaw cycles on dry ice. Samples were then incubated with 50 units/ml of lysozyme for 15 minutes. Next, physical disruption was performed in a Bead-Beater (BioSpec Products, Inc., Bartlesville, OK) on the homogenize setting for three minutes. An overnight incubation with 1% SDS and 2 mg/ml Proteinase K was followed by extraction with an equal volume of 25∶24∶1 phenol:chloroform:isoamyl alcohol. The aqueous phase was incubated at room temperature for 30 minutes with 2.5 volumes of 100% isopropanol and 0.1 volumes of 3 M sodium acetate before centrifugation at 16,000 g for 30 minutes at 4°C. The DNA pellet was washed with cold 70% ethanol and allowed to air dry before resuspension in TE (10 mM Tris-HCl pH 7.5, 1 mM EDTA.).

### 16S library creation and sequencing

Approximately 100 ng of DNA was used as template for small-subunit rDNA (16S) amplification. Bacterial universal primers 27F (5′- AGAGTTTGATCCTGGCTCAG) and 1492R (5′-GGTTACCTTGTTACGACTT) were used to amplify a ∼1450 bp fragment (Lane, 1991). These primers were chosen for three reasons. First, although they are not truly universal, they are specific to a region that is conserved in many groups of bacteria [94]. Second, they allow for the amplification of nearly the full length of the gene, therefore providing consistent comparisons to previous studies of 16S rDNA diversity [95]. Finally, both of these primers have been used in many similar surveys of bacterial diversity, including a previous study of bacterial diversity in *Drosophila melanogaster* [32]. Using these primers allows our results to be directly comparable to those previous studies. The PCR conditions were as follows: initial denaturation for 5 minutes at 95°C; 30 or 35 cycles at 95°C for 30 seconds, 55°C for 30 seconds, and 72°C for 2 minutes; final extension for 10 minutes at 72°C. These PCR conditions were used for all samples, with an annealing temperature of 55°C chosen from a temperature gradient study of 48°C to 58°C because it produced the maximum product yield. The 16S rDNA amplicons were cloned into the pCR4-*TOPO* vector using the *TOPO* TA Cloning Kit. Clones were transformed chemically into One Shot *TOP10* chemically competent *E. coli* cells or via electroporation into ElectroMAX DH10B *E. coli* cells (Invitrogen, Carlsbad, CA) and plated onto agar plates with X-gal and either 50 mg/mL Kanamycin or 50 mg/mL Ampicillin. Colony PCR (20 colonies) was used to verify a <10% insertless rate and ∼1.5 kb insert size. White colonies were arrayed into 384-well plates. Prior to sequencing, plasmids were amplified by rolling circle amplification using the TempliPhi DNA Sequencing Amplification Kit (Amersham Biosciences, Piscataway, NJ) and sequenced from both ends using the M13 (−28 or −40) primers with the BigDye kit (Applied Biosystems, Foster City, CA). Sequencing reactions were purified using magnetic beads and run on an ABI PRISM 3730 (Applied Biosystems) sequencing machine.

### Sequence quality assurance

Vector and primer sequences were removed with *cross_match*, a component of the *Phrap* software package [96], [97], and bases with a PHRED quality score of Q> = 15 were converted to “N”s using JAZZ, the Joint Genome Institute's in-house assembly algorithm. When possible, overlapping regions from the forward and reverse reads of each clone were used to assemble a single contiguous sequence for each clone. In cases where the overlap was not sufficient for assembly, custom perl scripts were used to concatenate the forward and reverse reads with gaps inserted between them (see below). All sequence data are available via BioTorrents (http://biotorrents.net/details.php?id=143) and have been submitted GenBank under the accession numbers JN420379 through JN426767.

### Sequence alignment

We used the *Infernal 1.0* software [98] to create a single multiple sequence alignment for all of our samples. *Infernal* creates a Hidden Markov Model (HMM) based on a high-quality reference alignment with a fixed length of 1532. 2078 of the 4198 clones consisted of non-overlapping paired reads; for those we created a 5′-alignment (of reads beginning with the 27F primer sequence) and a 3′-alignment (of reads ending with the reverse complement of the 1492R primer sequence), and merged the two alignments, inserting gaps into the intervening columns, based on positions in the reference alignment. The concatenated sequences from this “merged” alignment were combined with the successfully-assembled, full-length clones to create a single multiple sequence alignment. This alignment is available via BioTorrents (http://biotorrents.net/details.php?id=143).

For the purposes of OTU (operational taxonomic units [99]) definition and phylogenetic inference, this multiple sequence alignment was further refined to remove column blocks that contained >80% gaps. This resulted in the removal of the first 11 (1–11) and last 132 (1400–1532) positions, as well as positions 642–806 (164 positions) from the middle of the alignment (which primarily corresponded to the regions of non-overlap between reads). A custom perl script was used to remove sequences with fewer than 300 remaining nucleotides from this trimmed alignment. Chimeric sequences were identified and removed using the *chimera.slayer* function within *mothur v.1.11* [35].

### Taxonomy prediction and OTU assignment

We submitted our sequences to the Ribosomal Database Project (RDP10) Classifier for taxonomic assignment [100] to the genus level. We were unable to submit a single, full-length sequence for every 16S clone that was sequenced, because for 50% of our clones, there was no overlap between the forward and reverse reads. For each clone, we assigned taxonomy independently to the 5′ read, the 3′ read, and to the full-length or concatenated reads (with intervening gaps inserted, as described above), and then selected a single taxonomy assignment for each 16S clone. We used the measures of confidence (bootstrap values) that are associated with the RDP taxonomy predictions to guide the selection process. Most investigators agree that >70% bootstrap support is indicative of strong support for a phylogenetic clade [101]. In order to arrive at taxonomy predictions with very high confidence, we only considered taxonomy assignments that had bootstrap values of >75% at the genus level, >80% at the family level, >95% at the order level, and 100% at the class level. Strongly supported disagreements between the 5′, 3′, and combined data sets were rare (72 total sequences). These were handled in one of two ways: 1) if the conflict was at the level of family or above, they were considered likely to be chimeric sequences and excluded from further analysis, or 2) if the conflict was within a single family, the genus name was changed to “unclassified”.

We used the *mothur* program [35] to generate a distance matrix using our trimmed *Infernal* alignment of 3243 sequences as input. Using the distance matrix created by *mothur*, sequences were clustered using the average neighbor algorithm. Using the 0.03 cutoff option (97% sequence similarity), all sequences fell into 139 OTUs. The average OTU abundance was 23.3 sequences (Min = 1, Max = 638). A representative sequence from each OTU was selected using the *get.oturep* function within *mothur*. This representative sequence and the *dist.seqs* command in *mothur* was used to calculate genetic distances between OTUs and representative sequences throughout this study.

Taxonomy predictions generated by RDP were mapped onto each sequence within an OTU. In many cases, this led to a clear reassignment of “unclassified” sequences to the genus level based on the dominant genus present in that OTU. In other cases, the entire OTU was comprised of “unclassified” sequences. These OTUs were assigned names based on their phylogenetic position relative to the reference sequences included, either from the RDP type strains, from other *Drosophila* bacterial microbiome studies, or from GenBank.

### Representative sequences for tree building, PCA analysis, and diversity comparisons

All 7448 good quality 16S sequences longer than 1200 bp from bacterial type strains were downloaded from the RDP website on 8/22/10 [100]. These representatives are usually the first identified or most fully characterized strains within a bacterial lineage. Although closely related bacteria may differ substantially in genome content, inclusion of these type strains provides important phylogenetic landmarks during tree building. All 7448 strains were aligned using *Infernal,* and the resulting alignment was trimmed to remove the first 11 (1–11) and last 132 (1400–1532) positions, as well as positions 642–806 (164 positions) from the middle of the alignment as described above.

All sequences from previous studies of *Drosophila* bacterial communities [32], [33] (Corby-Harris, unpublished) were downloaded from GenBank. *Mothur* was used to create a distance matrix, and OTUs were created at a 97% similarity cutoff. The *get.oturep* function was used to pick a representative sequence for each OTU. Additional *Drosophila*-associated sequences were also included [21], [37]. Finally, for OTUs in our study that do not have any closely related sequences within the RDP database (such as *Enterobacteriaceae Group Orbus*) the closest BLAST hits from GenBank were included. A list of the RDP and GenBank accession numbers for sequences used in the final tree are found in the Dataset S5.

To compare our results to mammalian studies, the 17,504 ultraclean sequences from [12] were analyzed. To obtain a sample that was roughly equal to our data in taxonomic breadth, only the sequences from *Artiodactyla*, *Carnivora*, and *Primate* samples were analyzed. These sequences were aligned and trimmed as above and a full PCA analysis was performed using the *Fast UniFrac Interface* [62]. Taxonomic classifications were done with RDP [100].

Diversity measurements were calculated for each library from both *Drosophila* [33] and mammalian [12] datasets using *mothur* [35]. The proportion of OTUs unique to each library was calculated for each group (as in Ley et al., 2008a) [12].

### Tree building

Using representative sequences from our dataset, previously identified *Drosophila*-associated bacteria, representative type strains from the RDP database, and sequences obtained from GenBank (see previous section), a phylogenetic tree was created with *FastTree* [36]. Default settings with the GTR (generalized time-reversible) model were used. The entire tree was rooted using *Thermus thermophilus* (RDP identifier S000381199). After an initial run with all 8,407 sequences, many clades were removed from the alignment (for example, bacterial Phyla in which no *Drosophila* associated sequences were present). The remaining 1349 aligned sequences were then re-run on *FastTree* using the settings described above. Final publication quality images were prepared using *Dendroscope* [102].

### *UniFrac* significance tests

Tests of significance of differences between samples were performed using *UniFrac* and *FastUniFrac* [61], [62]. The low depth of coverage provided by the sequencing method used is sufficient to find significant results using *UniFrac* [103]. Because of the correction for multiple comparisons, pairwise comparisons for each library were not feasible with the amount of data collected. We therefore parsed all data into bins representing different host diets to estimate the overall effect of this factor. The effect of different experimental treatments was determined similarly. All comparisons of bacterial communities are given in the Text S1. Co-occurrence tests were also performed (as in [104]), but inadequate power precluded the finding of any significant co-occurring pairs of taxa (additional details in Text S1).

### Laboratory experiments

Unless explicitly stated, flies were fed unsterilized standard lab media (Text S1). All transfer steps were performed near a Bunsen burner flame and all surfaces and instruments were frequently sterilized with 70% ethanol. For bacterial DNA extraction, flies were washed to remove external bacterial cells and their intestines dissected as described above. All negative controls were confirmed to be bacteria-free by plating onto MRS media and PCR with universal bacterial primers. Separate libraries were created from adult Canton-S males, Oregon-R males, and Oregon-R females for Deborah Kimbrell's lab (UC-Davis, CA). In addition, a large population of wild *D. melanogaster* originally collected from Winters, CA was established in the Kopp lab for use in dietary treatment experiments (strain WO).

For diet experiments, approximately 25 flies were transferred to each of 5 separate diets, with one vial per treatment. The diets included standard lab media, high yeast media, high yeast supplemented with 6% ethanol, sugar-agar, and agar only (see Text S1 for media composition). All media were initially sterilized in an autoclave, with the exception of the standard lab media. Ethanol was added to the ethanol treatment after media cooled below 55°C. To reduce the effect of the media-dwelling bacterial population that arose after contact with non-sterile flies, cultures were transferred daily to fresh sterile media, with the exception of the standard lab diet. These transfers continued for three days on all media except the agar-only diet, where starvation-induced death limited the experiment to two days. Four hours after the final transfer, the intestines of 10 flies per treatment were dissected for analysis.

For the multiple species experiment, approximately 25 adults each of *D. melanogaster*, *D. elegans,* and *D. virilis* were combined on sterilized high yeast media. After three days of daily transfers as above, 10 males per species were dissected for analysis.

## Supporting Information

Dataset S1 A more detailed version of Table 1 describing where, when and by whom each sample was collected.
(XLS)

Dataset S2 This excel file contains the taxonomy assigned to each sequence used in this study along with information regarding the host species, location, environment and other information regarding the library each sequence belongs to. Additionally, the composition of each OTU can be determined using the unique OTU identifiers (used in Figures S1, S2, S3, S4, and S5 and in the main text).
(XLSX)

Dataset S3 This table describes the samples which *Wolbachia* and *Spiroplasma* were found in. Note that samples TRR and TUR were removed from the overall analysis because, after the removal of their *Wolbachia* sequences, they each were left with too few sequences for analysis. Each of these samples was a *D. melanogaster/D. simulans* mix collected from citrus fruit at Michael Turelli' orchard in Winters, California.
(XLSX)

Dataset S4 This file contains the beta-diversity measurements for all possible comparisons between our 20 natural *Drosophila* samples.
(XLSX)

Dataset S5 This file contains the GenBank accession numbers and the RDP identifiers of all the sequences used for tree building in this study.
(TXT)

Figure S1 Phylogenetic tree of the Enterobacteriaceae Group Orbus found with *Drosophila*. Taxa highlighted in red are OTUs identified within this study. Each OTU begins with a unique identifier corresponding to a sequence within the FASTA files available on BioTorrents (http://biotorrents.net/details.php?id=143). The number of libraries and the number of sequences each OTU represents is also given. For each, this is further divided into how many libraries/sequences were found in either laboratory or wild samples. For example, OTU HCF 018-#libs(2/10)-#seqs(3/389) represents 3 sequences found in 2 laboratory libraries and 389 sequences found in 10 wild libraries. Taxa highlighted in green are from previous studies of the bacterial communities associated with *Drosophila*. The taxon highlighted in purple is the cultured isolate that this group is named after (see main text). Unhighlighted taxa are type strains found within the Ribosomal Database Project (RDP) or taxa found in GenBank. Each of these taxa is followed by its GenBank accession number, its RDP identifier, or a unique identifier which corresponds to a sequence within the FASTA files available on BioTorrents (http://biotorrents.net/details.php?id=143). The orange edge in this figure corresponds to the orange node in Figure S2. The main phylogenetic tree of which this tree is a subset was rooted using *Thermus thermophilus* (RDP identifier S000381199).
(PDF)

Figure S2 Phylogenetic tree of the Enterobacteriaceae found with *Drosophila*. Taxa highlighted in red are OTUs identified within this study. Each OTU begins with a unique identifier corresponding to a sequence within the FASTA files available on BioTorrents (http://biotorrents.net/details.php?id=143). The number of libraries and the number of sequences each OTU represents is also given. For each, this is further divided into how many libraries/sequences were found in either laboratory or wild samples. For example, OTU HCF 018-#libs(2/10)-#seqs(3/389) represents 3 sequences found in 2 laboratory libraries and 389 sequences found in 10 wild libraries. Taxa highlighted in green are from previous studies of the bacterial communities associated with *Drosophila*. Unhighlighted taxa are type strains found within the Ribosomal Database Project (RDP). Each of these taxa is followed by its GenBank accession number, its RDP identifier, or a unique identifier which corresponds to a sequence within the FASTA files available on BioTorrents (http://biotorrents.net/details.php?id=143). The orange node in this figure corresponds to the orange edge in Figure S1. The main phylogenetic tree of which this tree is a subset was rooted using *Thermus thermophilus* (RDP identifier S000381199).
(PDF)

Figure S3 Phylogenetic tree of the Acetobacteraceae found with *Drosophila*. Phylogenetic trees of bacterial groups associated with Drosophila. Taxa highlighted in red are OTUs identified within this study. Each OTU begins with a unique identifier corresponding to a sequence within the FASTA files available on BioTorrents (http://biotorrents.net/details.php?id=143). The number of libraries and the number of sequences each OTU represents is also given. For each, this is further divided into how many libraries/sequences were found in either laboratory or wild samples. For example, OTU HCF 018-#libs(2/10)-#seqs(3/389) represents 3 sequences found in 2 laboratory libraries and 389 sequences found in 10 wild libraries. Taxa highlighted in green are from previous studies of the bacterial communities associated with *Drosophila*. The taxon highlighted in purple is a cultured isolate that closely related OTUs from this study were named after (see main text). Unhighlighted taxa are type strains found within the Ribosomal Database Project (RDP). Each of these taxa is followed by its GenBank accession number, its RDP identifier, or a unique identifier which corresponds to a sequence within the FASTA files available on BioTorrents (http://biotorrents.net/details.php?id=143). The main phylogenetic tree of which this tree is a subset was rooted using *Thermus thermophilus* (RDP identifier S000381199).
(PDF)

Figure S4 Phylogenetic tree of the Lactobacilli found with *Drosophila*. Taxa highlighted in red are OTUs identified within this study. Each OTU begins with a unique identifier corresponding to a sequence within the FASTA files available on BioTorrents (http://biotorrents.net/details.php?id=143). The number of libraries and the number of sequences each OTU represents is also given. For each, this is further divided into how many libraries/sequences were found in either laboratory or wild samples. For example, OTU HCF 018-#libs(2/10)-#seqs(3/389) represents 3 sequences found in 2 laboratory libraries and 389 sequences found in 10 wild libraries. Taxa highlighted in green are from previous studies of the bacterial communities associated with *Drosophila*. Unhighlighted taxa are type strains found within the Ribosomal Database Project (RDP). Each of these taxa is followed by its GenBank accession number, its RDP identifier, or a unique identifier which corresponds to a sequence within the FASTA files available on BioTorrents (http://biotorrents.net/details.php?id=143). The main phylogenetic tree of which this tree is a subset was rooted using *Thermus thermophilus* (RDP identifier S000381199).
(PDF)

Figure S5 Phylogenetic tree of the Enterococci found with *Drosophila*. Taxa highlighted in red are OTUs identified within this study. Each OTU begins with a unique identifier corresponding to a sequence within the FASTA files available on BioTorrents (http://biotorrents.net/details.php?id=143). The number of libraries and the number of sequences each OTU represents is also given. For each, this is further divided into how many libraries/sequences were found in either laboratory or wild samples. For example, OTU HCF 018-#libs(2/10)-#seqs(3/389) represents 3 sequences found in 2 laboratory libraries and 389 sequences found in 10 wild libraries. Taxa highlighted in green are from previous studies of the bacterial communities associated with *Drosophila*. Unhighlighted taxa are type strains found within the Ribosomal Database Project (RDP). Each of these taxa is followed by its GenBank accession number, its RDP identifier, or a unique identifier which corresponds to a sequence within the FASTA files available on BioTorrents (http://biotorrents.net/details.php?id=143). The main phylogenetic tree of which this tree is a subset was rooted using *Thermus thermophilus* (RDP identifier S000381199).
(PDF)

Figure S6 Number of OTUs as a function of genetic distance. Number of OTUs was calculated at all genetic distances from 0 (unique sequences) to 0.37 (the largest distance between any two sequences). Clustering was performed using the average neighbor algorithm in *mothur* [35].
(TIF)

Figure S7 Proportion of OTUs that are unique to a single library for wild *Drosophila* and mammals. Calculations done as in Ley et al., 2008a [12].
(TIF)

Table S1 Diversity of bacterial communities associated with wild flies.
(DOC)

Table S2 Diversity of bacterial communities associated with laboratory samples.
(DOC)

Table S3 Comparison of the wild *Drosophila* samples in this and previous studies.
(DOC)

Table S4 Taxonomic comparison of the dominant bacterial orders found within *Drosophila* and mammals.
(DOC)

Table S5 Gut bacterial microbiome composition in *D. melanogaster* strains from different labs.
(DOC)

Table S6 Variation in *D. melanogaster* bacterial microbiome on rich media at different times within the same laboratory.
(DOC)

Table S7 Comparison between internal and external bacterial microbiome of *Drosophila* adults (August, 2008) and larvae (July, 2008).
(DOC)

Text S1 The composition of all the laboratory diets used in this study are described here along with which libraries are included for each *UniFrac* comparison. Additionally, a description of the co-occurrence tests that were attempted is included.
(DOC)
