# Supplementary figures and images for: Bacterial Communities of Diverse Drosophila Species: Ecological Context of a Host–Microbe Model System
Source: PLoS Genet. 2011 Sep 22;7(9):e1002272. doi: 10.1371/journal.pgen.1002272 (PMC3178584; doi:10.1371/journal.pgen.1002272)

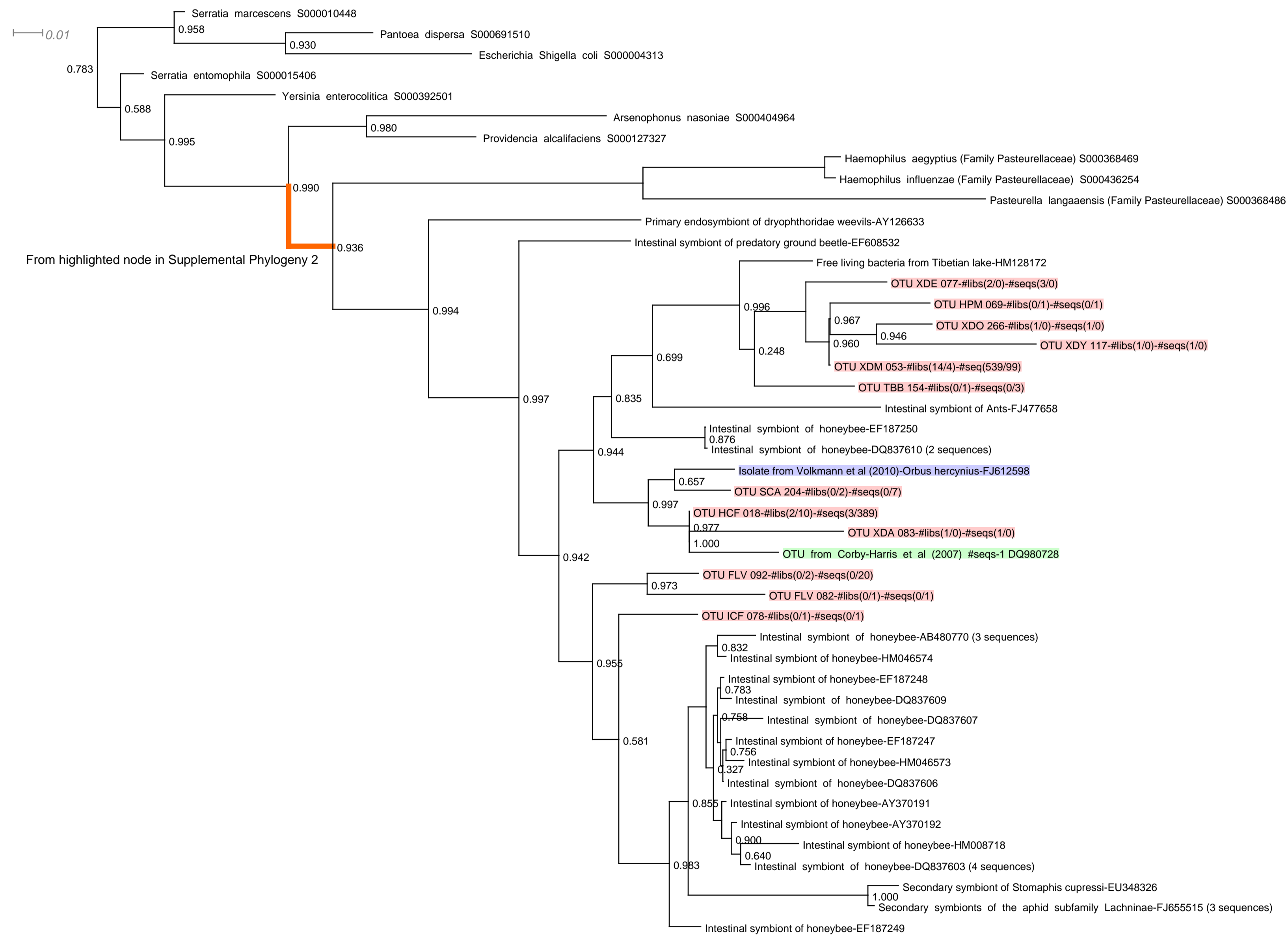

Supplement: Figure S1 — Phylogenetic tree of the Enterobacteriaceae Group Orbus found with Drosophila. Taxa highlighted in red are OTUs identified within this study. Each OTU begins with a unique identifier corresponding to a sequence within the FASTA files available on BioTorrents (http://biotorrents.net/details.php?id=143). The number of libraries and the number of sequences each OTU represents is also given. For each, this is further divided into how many libraries/sequences were found in either laboratory or wild samples. For example, OTU HCF 018-#libs(2/10)-#seqs(3/389) represents 3 sequences found in 2 laboratory libraries and 389 sequences found in 10 wild libraries. Taxa highlighted in green are from previous studies of the bacterial communities associated with Drosophila. The taxon highlighted in purple is the cultured isolate that this group is named after (see main text). Unhighlighted taxa are type strains found within the Ribosomal Database Project (RDP) or taxa found in GenBank. Each of these taxa is followed by its GenBank accession number, its RDP identifier, or a unique identifier which corresponds to a sequence within the FASTA files available on BioTorrents (http://biotorrents.net/details.php?id=143). The orange edge in this figure corresponds to the orange node in Figure S2. The main phylogenetic tree of which this tree is a subset was rooted using Thermus thermophilus (RDP identifier S000381199). (PDF) [file pgen.1002272.s006.pdf]

0.01

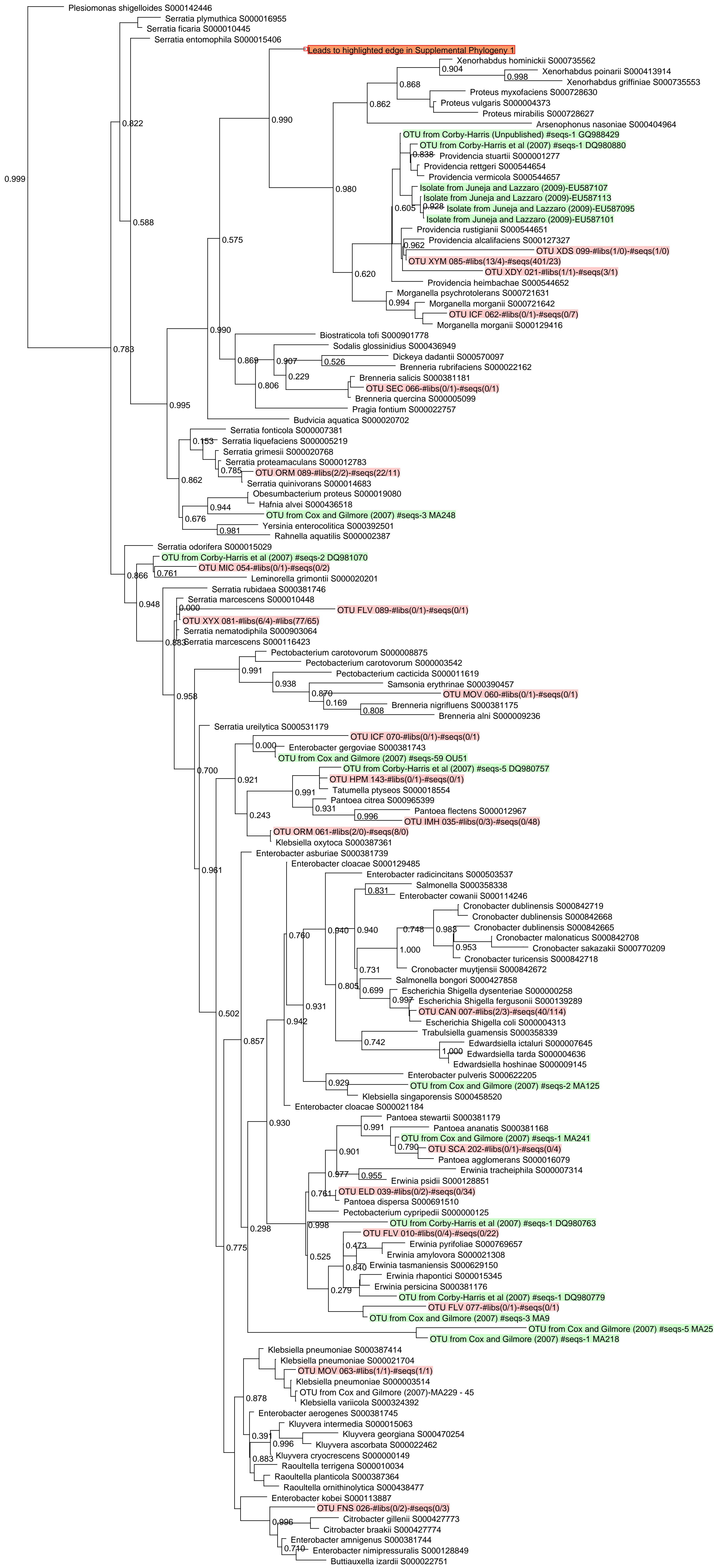

Supplement: Figure S2 — Phylogenetic tree of the Enterobacteriaceae found with Drosophila. Taxa highlighted in red are OTUs identified within this study. Each OTU begins with a unique identifier corresponding to a sequence within the FASTA files available on BioTorrents (http://biotorrents.net/details.php?id=143). The number of libraries and the number of sequences each OTU represents is also given. For each, this is further divided into how many libraries/sequences were found in either laboratory or wild samples. For example, OTU HCF 018-#libs(2/10)-#seqs(3/389) represents 3 sequences found in 2 laboratory libraries and 389 sequences found in 10 wild libraries. Taxa highlighted in green are from previous studies of the bacterial communities associated with Drosophila. Unhighlighted taxa are type strains found within the Ribosomal Database Project (RDP). Each of these taxa is followed by its GenBank accession number, its RDP identifier, or a unique identifier which corresponds to a sequence within the FASTA files available on BioTorrents (http://biotorrents.net/details.php?id=143). The orange node in this figure corresponds to the orange edge in Figure S1. The main phylogenetic tree of which this tree is a subset was rooted using Thermus thermophilus (RDP identifier S000381199). (PDF) [file pgen.1002272.s007.pdf]

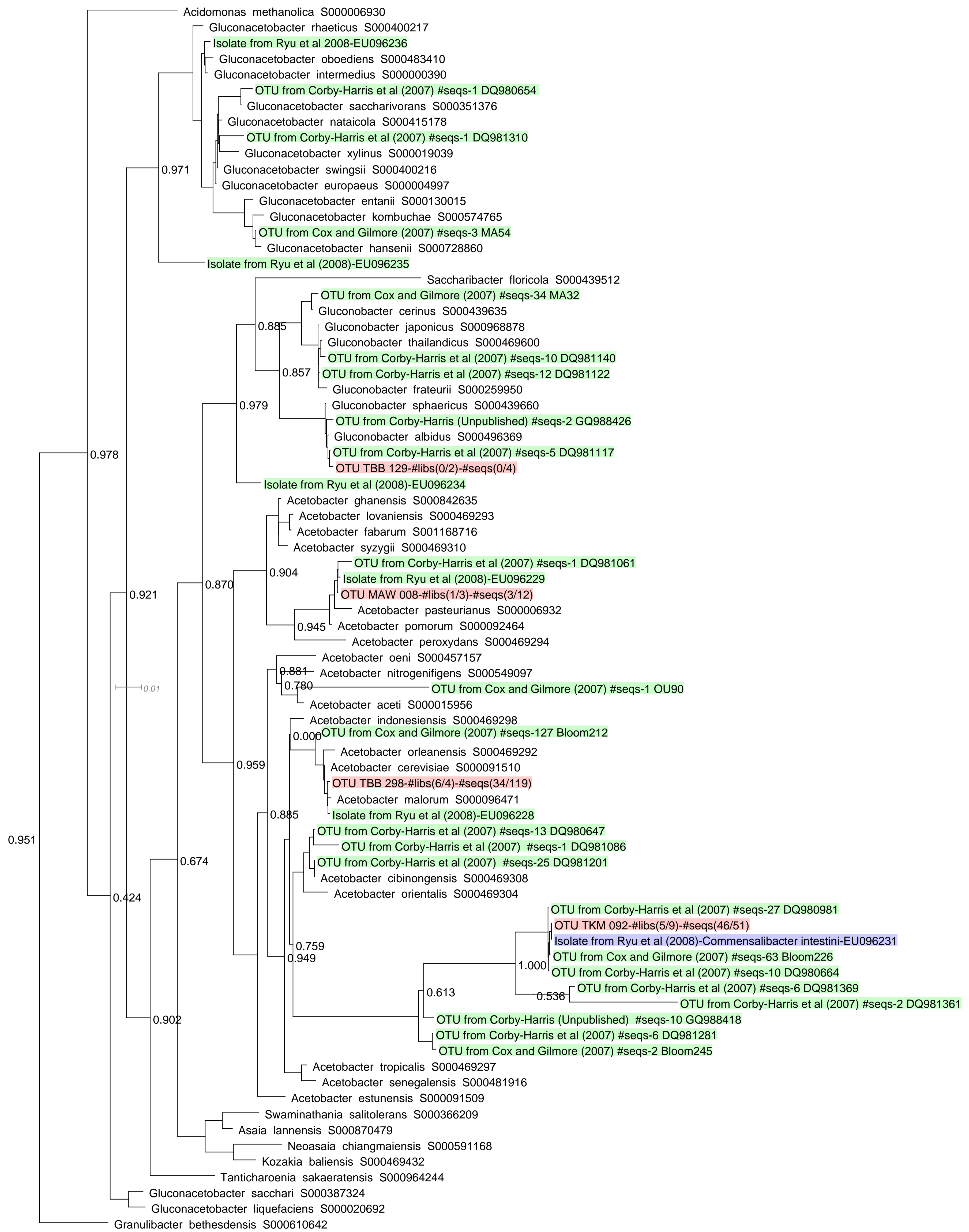

Supplement: Figure S3 — Phylogenetic tree of the Acetobacteraceae found with Drosophila. Phylogenetic trees of bacterial groups associated with Drosophila. Taxa highlighted in red are OTUs identified within this study. Each OTU begins with a unique identifier corresponding to a sequence within the FASTA files available on BioTorrents (http://biotorrents.net/details.php?id=143). The number of libraries and the number of sequences each OTU represents is also given. For each, this is further divided into how many libraries/sequences were found in either laboratory or wild samples. For example, OTU HCF 018-#libs(2/10)-#seqs(3/389) represents 3 sequences found in 2 laboratory libraries and 389 sequences found in 10 wild libraries. Taxa highlighted in green are from previous studies of the bacterial communities associated with Drosophila. The taxon highlighted in purple is a cultured isolate that closely related OTUs from this study were named after (see main text). Unhighlighted taxa are type strains found within the Ribosomal Database Project (RDP). Each of these taxa is followed by its GenBank accession number, its RDP identifier, or a unique identifier which corresponds to a sequence within the FASTA files available on BioTorrents (http://biotorrents.net/details.php?id=143). The main phylogenetic tree of which this tree is a subset was rooted using Thermus thermophilus (RDP identifier S000381199). (PDF) [file pgen.1002272.s008.pdf]

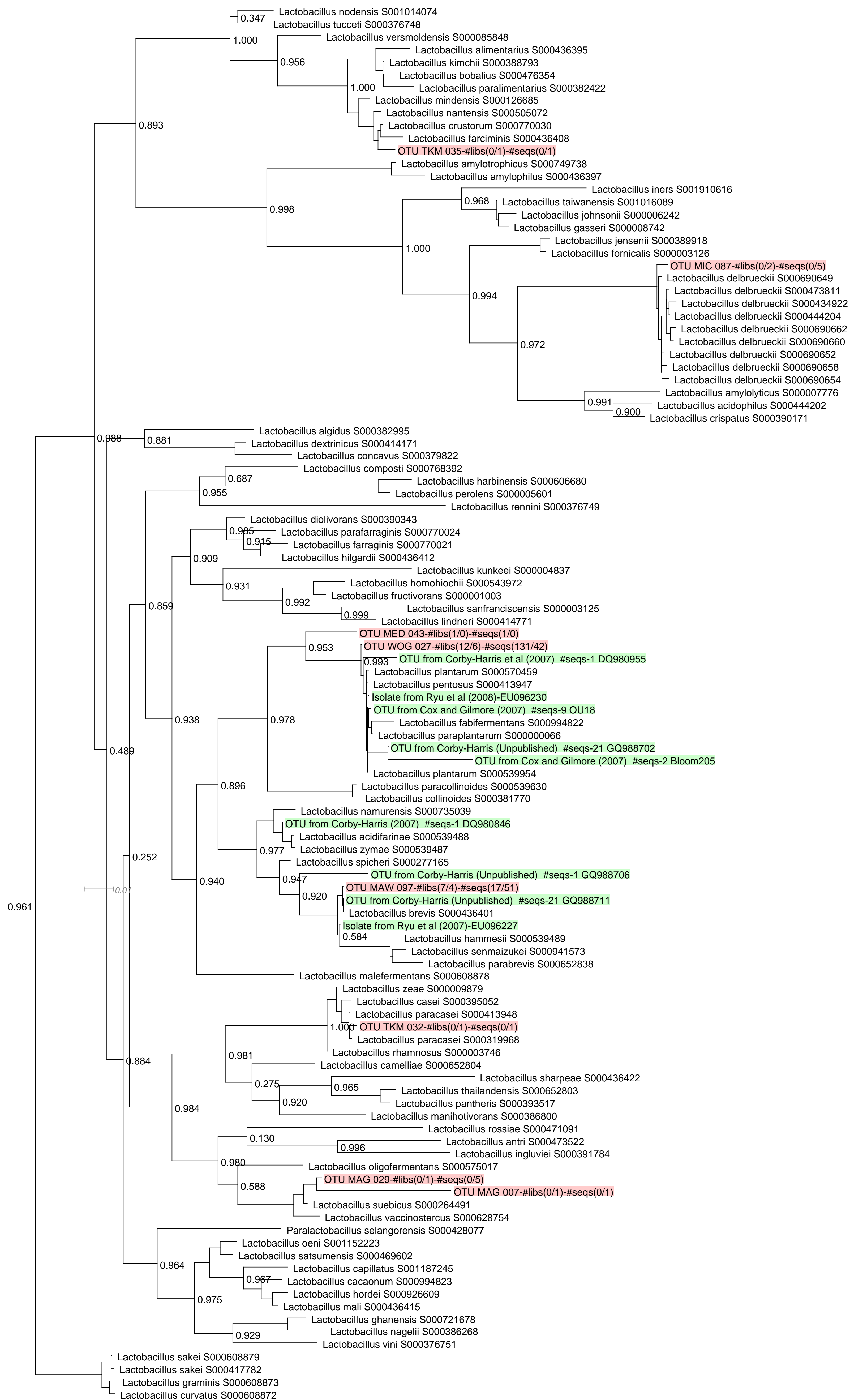

Supplement: Figure S4 — Phylogenetic tree of the Lactobacilli found with Drosophila. Taxa highlighted in red are OTUs identified within this study. Each OTU begins with a unique identifier corresponding to a sequence within the FASTA files available on BioTorrents (http://biotorrents.net/details.php?id=143). The number of libraries and the number of sequences each OTU represents is also given. For each, this is further divided into how many libraries/sequences were found in either laboratory or wild samples. For example, OTU HCF 018-#libs(2/10)-#seqs(3/389) represents 3 sequences found in 2 laboratory libraries and 389 sequences found in 10 wild libraries. Taxa highlighted in green are from previous studies of the bacterial communities associated with Drosophila. Unhighlighted taxa are type strains found within the Ribosomal Database Project (RDP). Each of these taxa is followed by its GenBank accession number, its RDP identifier, or a unique identifier which corresponds to a sequence within the FASTA files available on BioTorrents (http://biotorrents.net/details.php?id=143). The main phylogenetic tree of which this tree is a subset was rooted using Thermus thermophilus (RDP identifier S000381199). (PDF) [file pgen.1002272.s009.pdf]

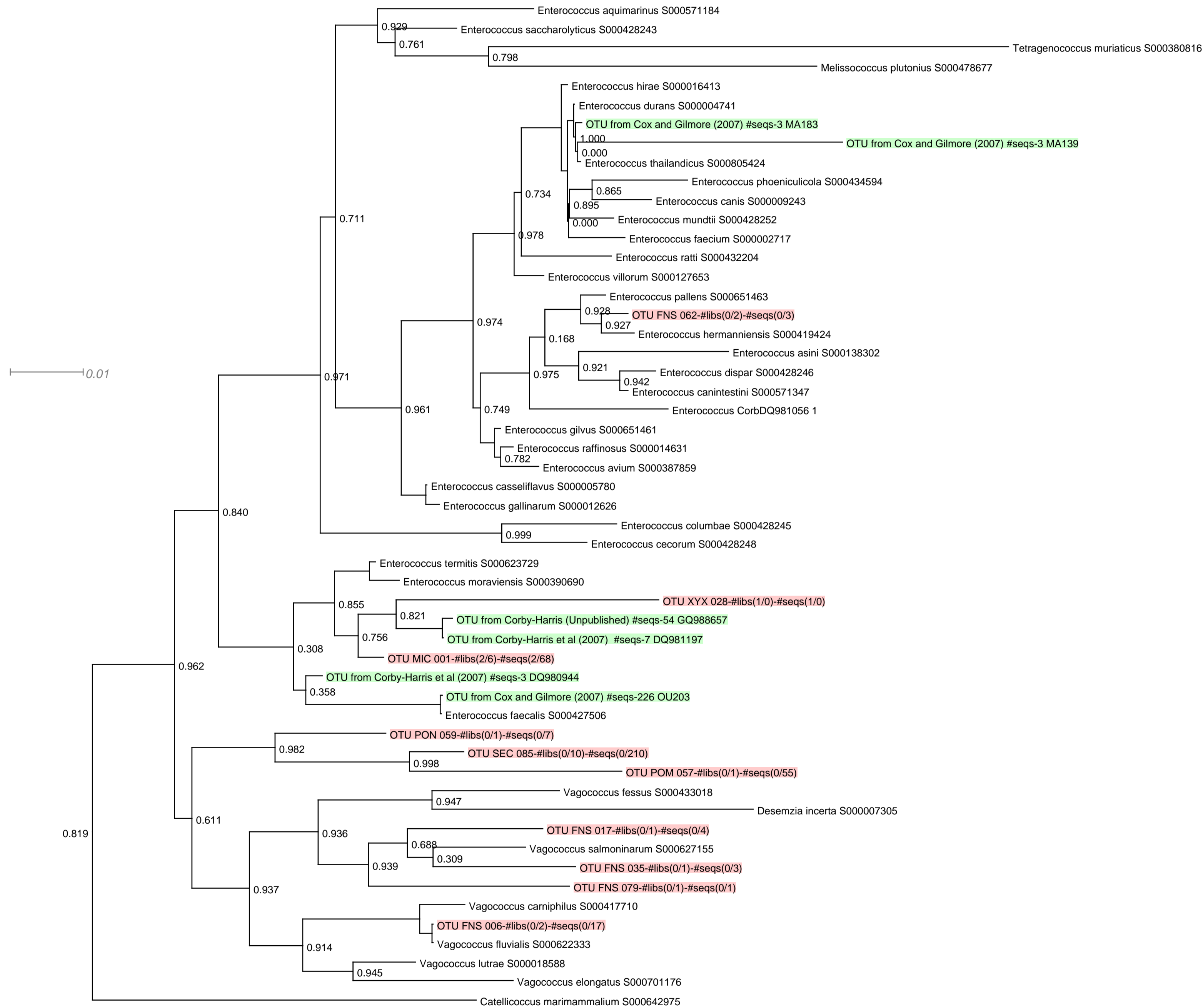

Supplement: Figure S5 — Phylogenetic tree of the Enterococci found with Drosophila. Taxa highlighted in red are OTUs identified within this study. Each OTU begins with a unique identifier corresponding to a sequence within the FASTA files available on BioTorrents (http://biotorrents.net/details.php?id=143). The number of libraries and the number of sequences each OTU represents is also given. For each, this is further divided into how many libraries/sequences were found in either laboratory or wild samples. For example, OTU HCF 018-#libs(2/10)-#seqs(3/389) represents 3 sequences found in 2 laboratory libraries and 389 sequences found in 10 wild libraries. Taxa highlighted in green are from previous studies of the bacterial communities associated with Drosophila. Unhighlighted taxa are type strains found within the Ribosomal Database Project (RDP). Each of these taxa is followed by its GenBank accession number, its RDP identifier, or a unique identifier which corresponds to a sequence within the FASTA files available on BioTorrents (http://biotorrents.net/details.php?id=143). The main phylogenetic tree of which this tree is a subset was rooted using Thermus thermophilus (RDP identifier S000381199). (PDF) [file pgen.1002272.s010.pdf]

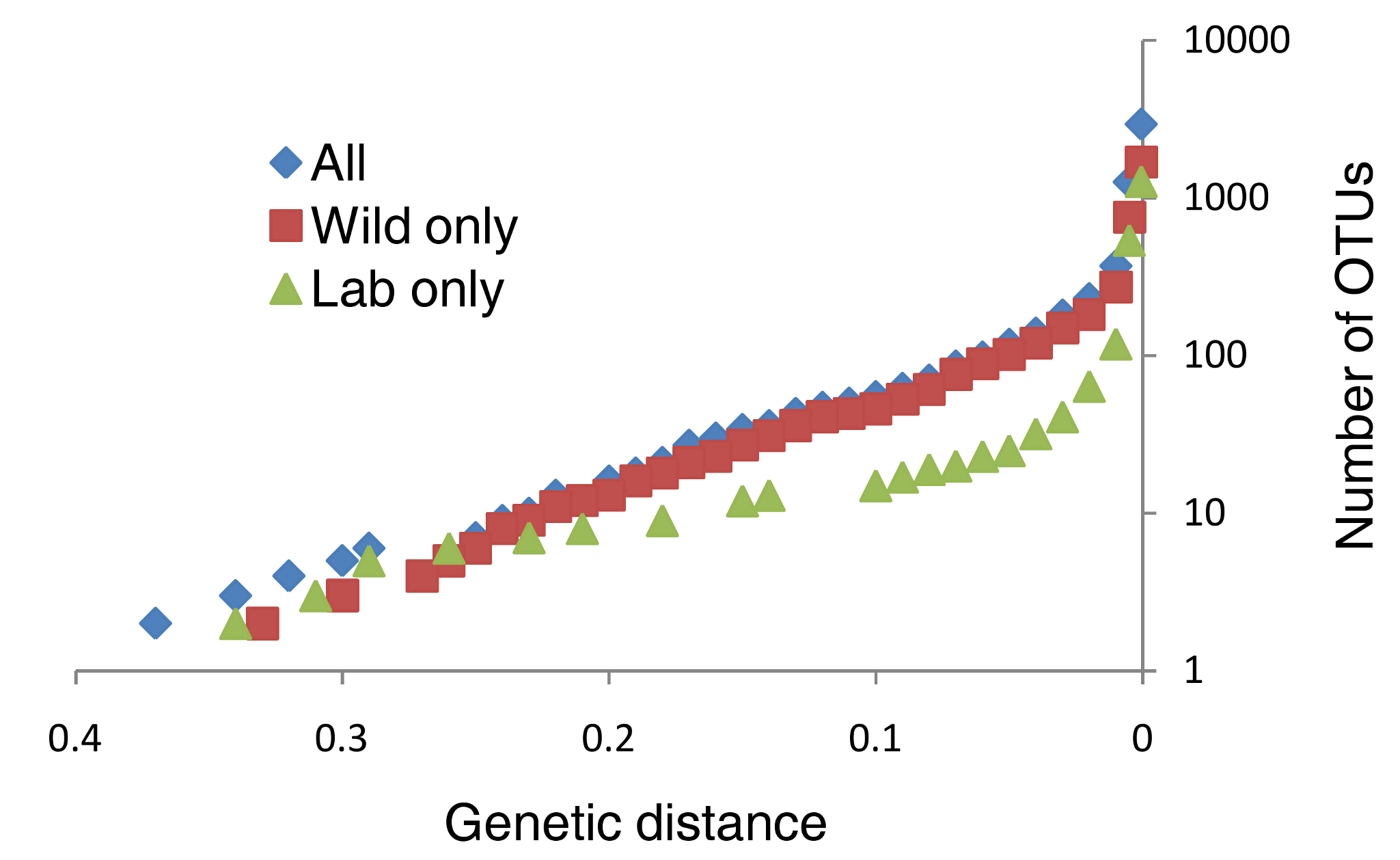

Supplement: Figure S6 — Number of OTUs as a function of genetic distance. Number of OTUs was calculated at all genetic distances from 0 (unique sequences) to 0.37 (the largest distance between any two sequences). Clustering was performed using the average neighbor algorithm in mothur [35]. (TIF) [file pgen.1002272.s011.tif]

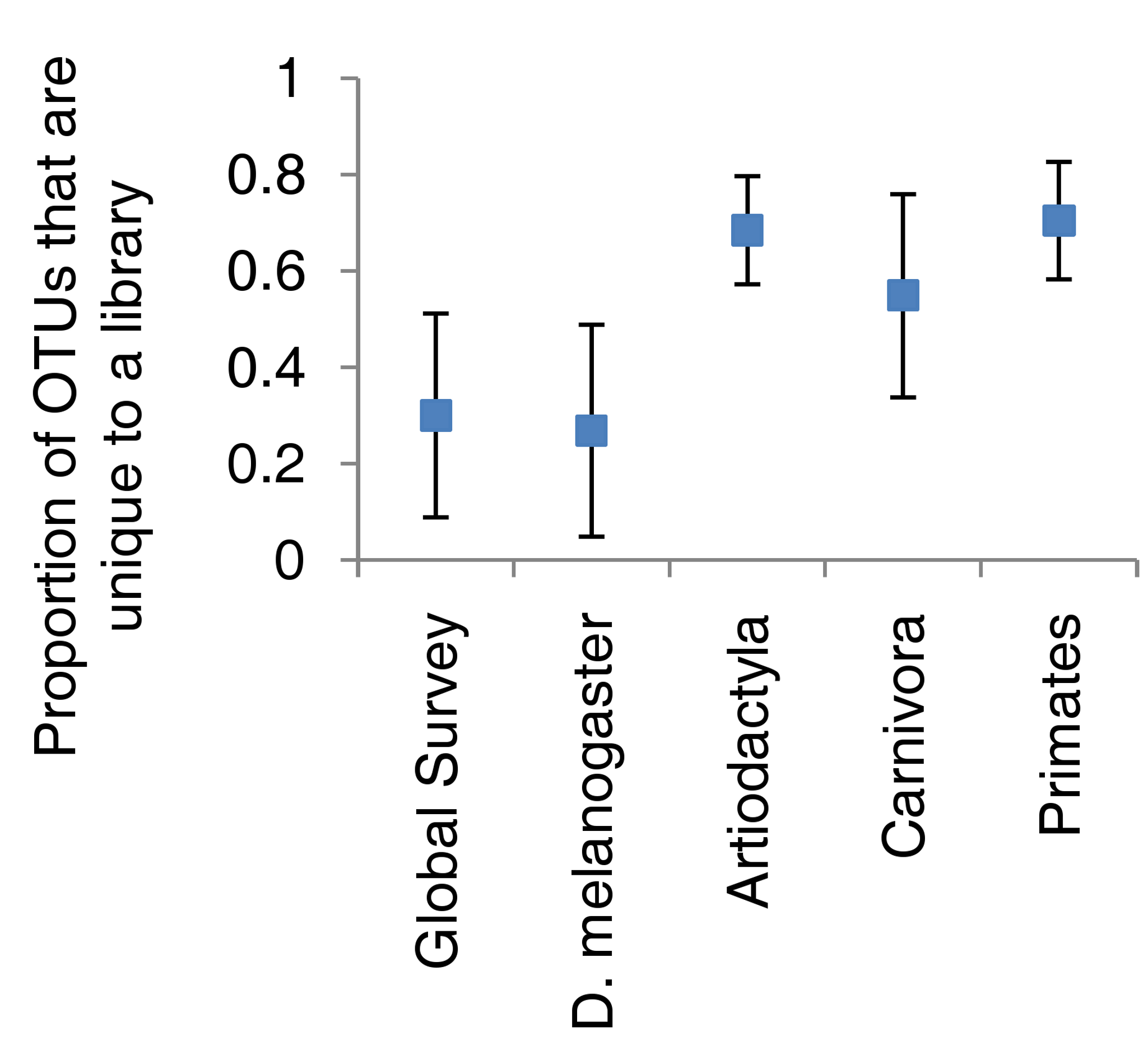

Supplement: Figure S7 — Proportion of OTUs that are unique to a single library for wild Drosophila and mammals. Calculations done as in Ley et al., 2008a [12]. (TIF) [file pgen.1002272.s012.tif]
