# Supplementary material for: Bacterial Communities of Diverse Drosophila Species: Ecological Context of a Host–Microbe Model System
Source: PLoS Genet. 2011 Sep 22;7(9):e1002272. doi: 10.1371/journal.pgen.1002272 (PMC3178584; doi:10.1371/journal.pgen.1002272)
Supplement: Table S1 — Diversity of bacterial communities associated with wild flies. (DOC) [file pgen.1002272.s013.doc]

| Library | Observed Richness  (# OTUs) | Good’s  Coverage | Chao1 Richness | Chao1 lci | Chao1 hci | Shannon Diversity | Shannon lci | Shannon hci | Shannon evenness |
| --- | --- | --- | --- | --- | --- | --- | --- | --- | --- |
| ELA | 20.00 | 0.91 | 23.00 | 20.55 | 36.46 | 2.48 | 2.25 | 2.70 | 0.83 |
| ELD | 7.00 | 0.97 | 7.50 | 7.03 | 15.26 | 1.32 | 1.11 | 1.53 | 0.68 |
| FLV | 11.00 | 0.90 | 18.50 | 12.32 | 53.53 | 1.69 | 1.44 | 1.94 | 0.71 |
| FNS | 30.00 | 0.75 | 57.20 | 38.26 | 119.53 | 3.04 | 2.80 | 3.28 | 0.89 |
| HCF | 4.00 | 1.00 | 4.00 | 4.00 | 4.00 | 0.54 | 0.33 | 0.75 | 0.39 |
| HPM | 17.00 | 0.93 | 50.00 | 25.28 | 148.50 | 1.34 | 1.15 | 1.54 | 0.47 |
| HPP | 3.00 | 0.99 | 3.00 | 3.00 | 0.00 | 0.32 | 0.15 | 0.49 | 0.29 |
| ICF | 9.00 | 0.97 | 9.75 | 9.07 | 17.45 | 1.22 | 0.97 | 1.47 | 0.55 |
| IMH | 7.00 | 0.97 | 8.50 | 7.15 | 22.08 | 1.07 | 0.87 | 1.26 | 0.55 |
| MAG | 6.00 | 0.95 | 12.00 | 6.97 | 43.22 | 0.44 | 0.19 | 0.69 | 0.24 |
| MAH | 4.00 | 1.00 | 4.00 | 4.00 | 4.00 | 0.89 | 0.74 | 1.04 | 0.64 |
| MAW | 5.00 | 0.99 | 5.00 | 5.00 | 0.00 | 1.24 | 1.09 | 1.40 | 0.77 |
| MIC | 15.00 | 0.92 | 18.75 | 15.64 | 37.02 | 1.70 | 1.39 | 2.02 | 0.63 |
| MOV | 9.00 | 0.94 | 12.33 | 9.50 | 31.07 | 0.76 | 0.47 | 1.05 | 0.35 |
| POM | 9.00 | 0.93 | 12.33 | 9.50 | 31.07 | 0.82 | 0.49 | 1.15 | 0.38 |
| PON | 12.00 | 0.91 | 22.50 | 14.03 | 66.19 | 1.48 | 1.20 | 1.76 | 0.59 |
| SCA | 26.00 | 0.90 | 30.00 | 26.86 | 44.61 | 2.90 | 2.72 | 3.09 | 0.89 |
| SEC | 5.00 | 0.98 | 6.00 | 5.07 | 18.50 | 0.62 | 0.40 | 0.83 | 0.38 |
| TBB | 16.00 | 0.98 | 26.00 | 17.90 | 68.70 | 1.86 | 1.70 | 2.02 | 0.67 |
| TKM | 11.00 | 0.89 | 12.20 | 11.14 | 21.37 | 1.85 | 1.47 | 2.22 | 0.77 |
| Average | 11.30 | 0.94 | 17.13 | 12.61 | 39.13 | 1.38 | 1.15 | 1.61 | 0.58 |
| SD | 7.42 | 0.06 | 14.75 | 9.19 | 38.41 | 0.77 | 0.77 | 0.78 | 0.20 |

All calculations were performed using *mothur* [35]. OTUs were defined at the 3% divergence threshold using the average neighbor clustering algorithm. Library identifiers are given in Table 1. lci=lower confidence interval; hci=higher confidence interval. Details regarding calculations can be found at <http://www.mothur.org/wiki/Calculators>.
