# Supplementary material for: Bacterial Communities of Diverse Drosophila Species: Ecological Context of a Host–Microbe Model System
Source: PLoS Genet. 2011 Sep 22;7(9):e1002272. doi: 10.1371/journal.pgen.1002272 (PMC3178584; doi:10.1371/journal.pgen.1002272)
Supplement: Table S2 — Diversity of bacterial communities associated with laboratory samples. (DOC) [file pgen.1002272.s014.doc]

| Library | Observed Richness  (# OTUs) | | Good’s  Coverage | | Chao1 Richness | | Chao1 lci | Chao1 hci | Shannon Diversity | Shannon lci | Shannon hci | Shannon evenness | |
| --- | --- | --- | --- | --- | --- | --- | --- | --- | --- | --- | --- | --- | --- |
| CAN | 3.00 | | 0.98 | | 3.00 | | 3.00 | 0.00 | 0.74 | 0.62 | 0.86 | 0.67 | |
| ORF | 7.00 | | 0.92 | | 7.50 | | 7.03 | 15.26 | 1.67 | 1.38 | 1.96 | 0.86 | |
| ORM | 8.00 | | 0.96 | | 9.00 | | 8.07 | 21.88 | 1.68 | 1.45 | 1.90 | 0.81 | |
| WOB | 5.00 | | 1.00 | | 5.00 | | 5.00 | 5.00 | 1.42 | 1.29 | 1.54 | 0.88 | |
| WOE | 5.00 | | 0.99 | | 5.00 | | 5.00 | 0.00 | 1.05 | 0.87 | 1.22 | 0.65 | |
| WOG | 3.00 | | 1.00 | | 3.00 | | 3.00 | 3.00 | 0.74 | 0.63 | 0.86 | 0.68 | |
| WOL | 1.00 | | 1.00 | | 1.00 | | 1.00 | 1.00 | 0.00 | 0.00 | 0.00 | 1.00 | |
| WOP | 6.00 | | 0.96 | | 6.33 | | 6.02 | 11.96 | 0.72 | 0.41 | 1.03 | 0.40 | |
| MED | 6.00 | | 0.94 | | 12.00 | | 6.97 | 43.22 | 0.47 | 0.21 | 0.74 | 0.26 | |
| Diet experiment (details in text) | | | | | | | | | | |  | | |
| XDA | | 7.00 | 0.97 | 7.33 | | 7.02 | | 12.96 | 1.35 | 1.16 | 1.55 | 0.69 |  |
| XDE | | 4.00 | 0.97 | 5.00 | | 4.08 | | 17.27 | 0.51 | 0.29 | 0.73 | 0.37 |  |
| XDM | | 2.00 | 1.00 | 2.00 | | 2.00 | | 2.00 | 0.33 | 0.19 | 0.47 | 0.48 |  |
| XDE | | 4.00 | 0.97 | 5.00 | | 4.08 | | 17.27 | 0.51 | 0.29 | 0.73 | 0.37 |  |
| XDO | | 6.00 | 0.98 | 12.00 | | 6.97 | | 43.22 | 0.59 | 0.45 | 0.73 | 0.33 |  |
| XDS | | 4.00 | 0.96 | 7.00 | | 4.37 | | 28.09 | 0.20 | 0.02 | 0.38 | 0.14 |  |
| XDY | | 6.00 | 0.94 | 7.00 | | 6.07 | | 19.66 | 1.31 | 1.01 | 1.62 | 0.73 |  |
| Host species experiment (details in text) | | | | | | | | | |  | | | |
| XYM | | 6.00 | 0.98 | 7.00 | | 6.07 | | 19.66 | 0.97 | 0.74 | 1.21 | 0.54 |  |
| XYE | | 4.00 | 0.98 | 5.00 | | 4.08 | | 17.27 | 0.27 | 0.08 | 0.45 | 0.19 |  |
| XYV | | 3.00 | 0.97 | 3.00 | | 3.00 | | 0.00 | 0.33 | 0.05 | 0.60 | 0.30 |  |
| XYX | | 6.00 | 0.95 | 12.00 | | 6.97 | | 43.22 | 0.68 | 0.45 | 0.92 | 0.38 |  |
| All of the above samples | | | | | | | | | | |  | | |
| Average | | 4.80 | 0.97 | 6.21 | | 4.99 | | 16.10 | 0.78 | 0.58 | 0.97 | 0.54 |  |
| SD | | 1.82 | 0.02 | 3.21 | | 1.95 | | 14.40 | 0.49 | 0.47 | 0.53 | 0.25 |  |

All calculations were performed using *mothur* [35]. OTUs were defined at the 3% divergence threshold using the average neighbor clustering algorithm. Library identifiers are given in Table 1. lci=lower confidence interval; hci=higher confidence interval. Details regarding calculations can be found at <http://www.mothur.org/wiki/Calculators>.
