# Supplementary material for: Bacterial Communities of Diverse Drosophila Species: Ecological Context of a Host–Microbe Model System
Source: PLoS Genet. 2011 Sep 22;7(9):e1002272. doi: 10.1371/journal.pgen.1002272 (PMC3178584; doi:10.1371/journal.pgen.1002272)
Supplement: Table S3 — Comparison of the wild Drosophila samples in this and previous studies. (DOC) [file pgen.1002272.s015.doc]

|  | Global Survey | Southeast USA | Massachusetts | Sonoran Desert |
| --- | --- | --- | --- | --- |
| Acetobacteraceae | 0.10 | 0.43 | 0.50 | 0.04 |
| Enterobacteriaceae | 0.47 | 0.06 | 0.40 | 0.00 |
| Enterococcaceae | 0.20 | 0.04 | 0.03 | 0.18 |
| Lactobacillaceae | 0.06 | 0.01 | 0.00 | 0.09 |
| Leuconostocaceae | 0.02 | 0.16 | 0.01 | 0.49 |
| Staphylococcaceae | 0.00 | 0.00 | 0.00 | 0.17 |
| Other Taxa | 0.15 | 0.30 | 0.05 | 0.03 |
| Total number of Sequences | 1850 | 283 | 149 | 306 |
| Total number of Populations Sampled | 20 | 11 | 1 | 4 |

Massachusetts data is from Cox and Gilmore, 2007; Southeast USA data is from Corby-Harris et al., 2007; Sonoran Desert data is Corby-Harris, unpublished.
