# Supplementary material for: Bacterial Communities of Diverse Drosophila Species: Ecological Context of a Host–Microbe Model System
Source: PLoS Genet. 2011 Sep 22;7(9):e1002272. doi: 10.1371/journal.pgen.1002272 (PMC3178584; doi:10.1371/journal.pgen.1002272)
Supplement: Table S4 — Taxonomic comparison of the dominant bacterial orders found within Drosophila and mammals. (DOC) [file pgen.1002272.s016.doc]

|  | Global Survey | *D. melanogaster* | Artiodactyla | Carnivora | Primates |
| --- | --- | --- | --- | --- | --- |
| Actinomycetales | 0.00 | 0.00 | 0.14 | 0.01 | 0.00 |
| Bacteroidales | 0.01 | 0.01 | 0.14 | 0.01 | 0.28 |
| Clostridiales | 0.01 | 0.01 | 0.57 | 0.40 | 0.49 |
| Enterobacteriales | 0.47 | 0.06 | 0.00 | 0.25 | 0.01 |
| Lactobacillales | 0.29 | 0.22 | 0.02 | 0.20 | 0.08 |
| Rhodospirillales | 0.10 | 0.44 | 0.00 | 0.00 | 0.00 |
| Other Taxa | 0.11 | 0.26 | 0.12 | 0.12 | 0.14 |
| Total number of Sequences | 1850 | 283 | 3412 | 3868 | 4610 |
| Total number of Samples | 20 | 11 | 21 | 17 | 20 |
| Total number of Species | 14 | 1 | 13 | 9 | 17 |

*D. melanogaster* data is from Corby-Harris et al., 2007. Selected mammalian orders are from Ley et al., 2008a.
