# Supplementary material for: Bacterial Communities of Diverse Drosophila Species: Ecological Context of a Host–Microbe Model System
Source: PLoS Genet. 2011 Sep 22;7(9):e1002272. doi: 10.1371/journal.pgen.1002272 (PMC3178584; doi:10.1371/journal.pgen.1002272)
Supplement: Table S5 — Gut bacterial microbiome composition in D. melanogaster strains from different labs. (DOC) [file pgen.1002272.s017.doc]

|  | Kimbrell Lab | Kopp Lab |
| --- | --- | --- |
| *Acetobacter* | 0.06 | 0.00 |
| *Lactobacillus* | 0.01 | 0.13 |
| *Microbacterium* | 0.15 | 0.00 |
| *Providencia* | 0.08 | 0.28 |
| *Serratia* | 0.16 | 0.00 |
| *Shigella* | 0.28 | 0.00 |
| Unclassified Comamonadaceae*/Variovorax* | 0.17 | 0.00 |
| *Enterobacteriaceae Group Orbus* | 0.00 | 0.58 |
| Other Taxa | 0.08 | 0.01 |
| Total Number of Samples | 3 | 5 |
| Total Number of Sequences | 143 | 472 |

Note: The *Serratia* strain identified in the Kimbrell lab is different than the one in the Kopp lab (Supplementary Online Materials)
