# Supplementary material for: Bacterial Communities of Diverse Drosophila Species: Ecological Context of a Host–Microbe Model System
Source: PLoS Genet. 2011 Sep 22;7(9):e1002272. doi: 10.1371/journal.pgen.1002272 (PMC3178584; doi:10.1371/journal.pgen.1002272)
Supplement: Table S6 — Variation in D. melanogaster bacterial microbiome on rich media at different times within the same laboratory. (DOC) [file pgen.1002272.s018.doc]

|  | Initial Characterization | Diet Experiment |  |  | Species Experiment |
| --- | --- | --- | --- | --- | --- |
|  | Lab Media | Lab Media (Start) | Lab Media (3 days) | High Yeast | High Yeast |
| *Lactobacillus* | 0.66 | 0.01 | 0.00 | 0.03 | 0.01 |
| *Providencia* | 0.00 | 0.17 | 0.10 | 0.24 | 0.94 |
| *Enterobacteriaceae Group Orbus* | 0.34 | 0.82 | 0.90 | 0.65 | 0.01 |
| Other Taxa | 0.00 | 0.01 | 0.00 | 0.09 | 0.03 |
| Total Number of Sequences | 87 | 173 | 88 | 34 | 90 |
