# Supplementary material for: Bacterial Communities of Diverse Drosophila Species: Ecological Context of a Host–Microbe Model System
Source: PLoS Genet. 2011 Sep 22;7(9):e1002272. doi: 10.1371/journal.pgen.1002272 (PMC3178584; doi:10.1371/journal.pgen.1002272)
Supplement: Table S7 — Comparison between internal and external bacterial microbiome of Drosophila adults (August, 2008) and larvae (July, 2008). (DOC) [file pgen.1002272.s019.doc]

|  | August, 2008 | | | July, 2008 | |
| --- | --- | --- | --- | --- | --- |
|  | Externally Sterilized Bodies | Gut Contents | External Wash | Larvae | Media |
| Acetobacter | 0.00 | 0.00 | 0.30 | 0.00 | 0.00 |
| Commensalibacter | 0.27 | 0.00 | 0.03 | 0.00 | 0.00 |
| Lactobacillus plantarum | 0.15 | 0.63 | 0.57 | 0.00 | 0.01 |
| Lactobacillus brevis | 0.11 | 0.02 | 0.01 | 0.00 | 0.01 |
| Enterobacteriaceae Group Orbus | 0.40 | 0.34 | 0.09 | 1.00 | 0.90 |
| Providencia | 0.00 | 0.00 | 0.00 | 0.00 | 0.01 |
| Serratia | 0.00 | 0.00 | 0.00 | 0.00 | 0.04 |
| Other Taxa | 0.06 | 0.00 | 0.00 | 0.00 | 0.01 |
| Total Number of Sequences | 84 | 87 | 79 | 70 | 70 |
