# Supplementary material for: Bacterial Communities of Diverse Drosophila Species: Ecological Context of a Host–Microbe Model System
Source: PLoS Genet. 2011 Sep 22;7(9):e1002272. doi: 10.1371/journal.pgen.1002272 (PMC3178584; doi:10.1371/journal.pgen.1002272)
Supplement: Text S1 — The composition of all the laboratory diets used in this study are described here along with which libraries are included for each UniFrac comparison. Additionally, a description of the co-occurrence tests that were attempted is included. (DOC) [file pgen.1002272.s020.doc]

**Supplemental Recipes:**

All diets are in 100 ml water and 2% agarose

High Yeast Diet

16 grams yeast extract

4 grams sucrose

Sugar Only

20 grams sucrose

Ethanol

16 grams yeast extract

4 grams sucrose

6 ml ethanol added after cooled below 55 degrees C

Agar

(nothing added)

Standard Laboratory Media (Bloomington Recipe)

Total Volume 23 liters

Ingredients

Agar – 200 milliliters

23 Liters of Hot Water

Non-Active Yeast – 600 milliliters

Soy Flour – 500 milliliters

Corn Meal – 2100 milliliters

Malt Extract – 1475 milliliters

Corn Syrup, Light – 1.5 Liters

Acid Mix A (0.4% Propionic Acid and 0.06% Phosphoric Acid in water) – 225 milliliters

Tegosept Mix (38 grams Tegosept in 138 milliliters ethanol) – 130 milliliters

**Comparisons done with Unifrac:**

Comparison of fruit and flower feeding *Drosophila*:

Fruit feeding samples were-HCF, HPM, HPP, ICF, MAG, MAH, MAW, PON, SEC, TBB, TKM

Flower feeding samples were-ELA, ELD, FLV, IMH, POM, SCA

Overall effect of diet in laboratory experiment:

Samples used: XDA, XDE, XDO, XDS, XDY, XDM

Overall effect of host species in laboratory experiment:

Samples used: XYE, XYM, XYV

Comparison of internal and external communities for *D. melanogaster* adults:

Samples used: WOE, WOB, WOG

Comparison of internal larval communities and surrounding media:

Samples used: WOL, MED

Comparison between Kopp lab and Kimbrell lab (only used *D. melanogaster* samples on rich media):

Kopp lab samples were-WOG, XDM, XDO, XDY, XYM

Kimbrell lab samples were-CAN, ORF, ORM

Comparison of different sampling dates within the Kopp lab:

Date 1 sample: WOG

Date 2 samples: XDM, XDO, XDY

Date 3 sample: XYM

**Co-occurrence Tests:**

To analyze our data for specific co-occurrences between different microbial taxa, we performed a Fisher’s exact test between all possible pairs of microbes (as in ). The four cells in the contingency table denoted the number of samples containing both microbes, the number of samples containing microbe 1, the number of samples containing microbe 2, and the number of the samples containing neither. We did this analysis at four levels of taxonomic relatedness (OTU, genus, family and order). Because of the large number of comparisons at each level (for example, 630 for OTUs) relative to the small number of samples which these came from (20 Drosophila populations), finding significance becomes unlikely after p-value correction for multiple independent tests.

To confirm our inability to find significant co-occurrences with this dataset, we performed a power test. At the 0.05 significance level (i.e. even before correction for multiple independent tests) we find that power=0.38, or well below the accepted value of 0.80. Using our OTU level data, we find that a sample size greater than 72.5 is needed for significance after Bonferroni correction. Although this correction is quite strict, we conclude that we do not have the ability to find significant co-occurrences within this dataset.

1. Chaffron S, Rehrauer H, Pernthaler J, von Mering C (2010) A global network of coexisting microbes from environmental and whole-genome sequence data. Genome Research 20: 947-959.
